# Vancomycin‐Induced Acute Kidney Injury in Intensive Care Patients: A Target Trial Emulation Study Using Multicenter Routinely Collected Data

**DOI:** 10.1002/pds.70205

**Published:** 2025-08-28

**Authors:** Izak A. R. Yasrebi‐de Kom, Kitty J. Jager, Vianda S. Stel, Nicholas C. Chesnaye, Ameen Abu‐Hanna, Nicolette F. de Keizer, Dylan W. de Lange, Dave A. Dongelmans, Joanna E. Klopotowska, Giovanni Cinà, the RESCUE Study Group, C. S. C. Bouman, E. N. van Roon, J. ten Cate, P. F. Schutte, D. van Balen, S. Hendriks, C. Lau, W. J. Vermeijden, A. Beishuizen, J. B. Masselink, P. E. Spronk, H. J. M. van Kan, P. W. de Feiter, E.‐J. Wils, A. Wieringa, A. J. Valkenburg, W. M. van den Bergh, M. H. Renes, W. Bult, E. de Jonge, M. Hoeksema, E. J. Wesselink, I. M. Purmer, B. E. Bosma, S. H. W. van Bree, H. J. W. Lammers, R. J. Bosman, E. J. F. Franssen, A. Karakus, M. Sigtermans, E. M. Kuck

**Affiliations:** ^1^ Department of Medical Informatics Amsterdam University Medical Center Amsterdam the Netherlands; ^2^ Amsterdam Public Health Amsterdam the Netherlands; ^3^ ERA Registry, Department of Medical Informatics Amsterdam University Medical Center Amsterdam the Netherlands; ^4^ Department of Intensive Care and Dutch Poison Information Center University Medical Center Utrecht Utrecht the Netherlands; ^5^ Department of Intensive Care Medicine Amsterdam University Medical Center Amsterdam the Netherlands; ^6^ Institute for Logic, Language and Computation University of Amsterdam Amsterdam the Netherlands

**Keywords:** acute kidney injury, adverse drug event, intensive care, target trial emulation, vancomycin

## Abstract

**Purpose:**

The potential of vancomycin to cause acute kidney injury (AKI) in adult intensive care patients is subject to debate due to suboptimal designs of past studies. Therefore, we aimed to estimate the effect of initiating vancomycin versus one of several minimally nephrotoxic alternative antibiotics on the 14‐day risk of AKI using the target trial emulation framework.

**Methods:**

A hypothetical trial was emulated using routinely collected data from 15 Dutch intensive care units (ICUs) spanning 2010–2019. We used an active comparator control group with the following alternative antibiotics: clindamycin, linezolid, teicoplanin, meropenem, cefazolin, and daptomycin. AKI was diagnosed according to the KDIGO serum creatinine (SCr) criteria. Cumulative incidence curves were estimated using the Aalen–Johansen method and adjusted for confounding and selection bias through inverse probability of treatment and censoring weighting. Given the time lag of 24–48 h between changes in renal function and SCr, we summarized the estimates by calculating the absolute risks and risk differences at both 2 and 14 days after initiation.

**Results:**

We included 1809 ICU admissions. After adjustment, vancomycin was associated with a higher risk of AKI at 14 days of follow‐up compared to the alternative antibiotics (0.28 [95% confidence interval (CI) 0.21–0.34] vs. 0.17 [95% CI 0.14–0.20]; risk difference 0.11 [95% CI 0.04–0.19]), but not at 2 days of follow‐up (0.10 [95% CI 0.06–0.12] vs. 0.10 [95% CI 0.08–0.11]; risk difference 0.00 [95% CI −0.03–0.03]).

**Conclusions:**

Our findings indicate that vancomycin causes a higher risk of AKI compared to the alternative antibiotics. We recommend clinicians to be compliant with vancomycin‐induced AKI prevention strategies, such as therapeutic drug monitoring or the consideration of an alternative antibiotic if possible.

**Summary**

- The potential of vancomycin to cause acute kidney injury (AKI) in adult intensive care patients is subject to debate.
- We estimated the effect of initiating vancomycin versus one of several minimally nephrotoxic alternative antibiotics on 14‐day risk of AKI using the target trial emulation framework.
- The estimates were adjusted for confounding and selection bias through inverse probability of treatment and censoring weighting.
- Vancomycin was associated with a higher risk of AKI compared to the alternative antibiotics.
- We recommend clinicians to be compliant with vancomycin‐induced AKI prevention strategies, such as therapeutic drug monitoring or the consideration of an alternative antibiotic if possible.

## 1 Introduction

Acute kidney injury (AKI) is a frequent syndrome in intensive care unit (ICU) patients and is often caused by drugs [1, 2, 3, 4]. Optimizing pharmacotherapy may reduce the risk of drug‐induced AKI and associated negative outcomes [2, 5, 6]. One of the actively debated drugs regarding its potential to cause AKI is vancomycin [7, 8, 9].

Previous randomized controlled trials (RCTs) provided limited data on vancomycin‐induced AKI. A recent meta‐analysis included seven RCTs comparing vancomycin with alternative antibiotics and found a significantly increased risk of AKI due to vancomycin with moderate strength of evidence (risk ratio [RR] 2.45) [10]. However, none of these RCTs were designed to investigate the risk of AKI; all studies focused on hospitalized patients, and two studies excluded patients with impaired kidney function. In most RCTs, a balanced distribution of relevant baseline patient characteristics (e.g., renal function) was not confirmed or—in one RCT—not the case despite randomization [10, 11]. Furthermore, renal endpoints were not pre‐specified and, in some studies, not strictly defined, but assessed qualitatively [9, 10]. Although RCTs are the gold standard for studying intended effects, the study of side effects may be hindered by stringent eligibility criteria, limited sample size, and unanticipated analyses of endpoints, possibly leading to biased estimates [12, 13]. We therefore additionally need observational studies to investigate causal effects [12].

However, a meta‐analysis of past observational studies revealed that such studies often focused on the association between vancomycin blood concentration levels and AKI, and most of them suffered from a moderate to high risk of bias [14]. A few observational studies investigated vancomycin‐induced AKI in the ICU through the analysis of drug administrations [15, 16, 17, 18]. These studies were also limited, either by small sample sizes or the use of a control group consisting of non‐users. The latter approach is at risk of confounding bias, which could be addressed by utilizing an active comparator design [19, 20]. Furthermore, none of the observational studies applied the target trial emulation (TTE) framework, which has been shown to aid in preventing bias, including immortal time bias and prevalent user bias [21, 22, 23, 24].

In this study, we therefore applied the TTE framework to estimate the causal effect of initiating vancomycin versus one of several minimally nephrotoxic alternative antibiotics on the 14‐day risk of AKI in adult non‐dialysis dependent AKI‐free ICU admissions.

## 2 Methods

### 2.1 Study Design and Data Collection

**FIGURE 1:**
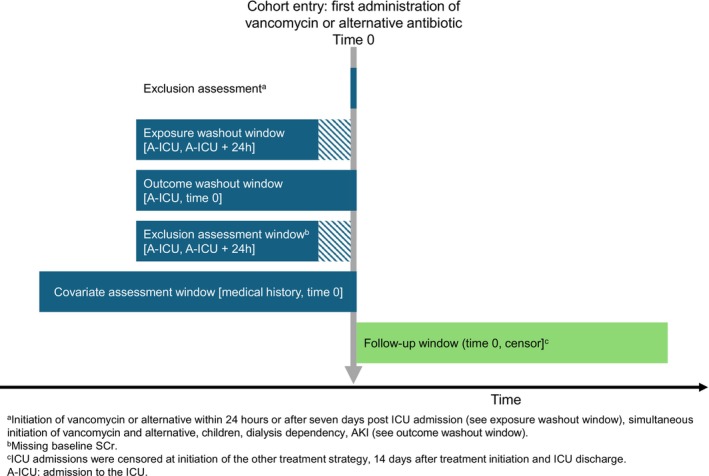
Graphical depiction of the study design. The gray line represents time zero. Areas with diagonal blue lines represent periods with varying durations across ICU admissions, as time zero could have started anywhere between 24 h and 7 days post ICU admission, whilst the preceding exposure washout window and exclusion assessment window ended at 24 h after admission to the ICU.

This TTE study employed an active comparator, new user design [20]. We re‐used routinely collected electronic health record (EHR) data of admissions to 15 Dutch ICUs between January 2010 and December 2019, and linked these records to data from the Dutch National Intensive Care Evaluation quality registry (NICE) [25]. We report our study according to the REporting of studies Conducted using Observational Routinely collected health Data for pharmacoepidemiology (RECORD‐PE) guideline (Supplementary Data S1) [26]. Supplementary methods are provided in Supplementary Data S2. See Table 1 for a glossary of terms and Figure 1 for a graphical depiction of the study design. For in‐depth explanations of the TTE framework, we refer to two methodological papers [21, 24].

**TABLE 1:** Glossary of terms.

Term	Meaning
Target trial emulation	Framework to design a hypothetical randomized controlled trial and emulate it using observational data. Usage aids in the identification and prevention of potential biases in causal inference using observational data.
Active comparator, new user study design	Study design that only includes new users of a drug and uses a control group with one or multiple alternative drugs. Decreases the risk of prevalent user bias and confounding.
Confounding	Bias caused by factors that are common causes of the exposure and the outcome.
Selection bias	Bias caused by conditioning on common effects of the exposure (or causes of the exposure) and the outcome (or causes of the outcome).
Prevalent user bias	A type of selection bias due to the start of follow‐up after initiation of a drug. Subjects in which the drug caused an outcome event shortly after initiation are not included and thus these outcome events are missed.
Immortal time bias	Bias caused by the assignment of treatment groups based on information after baseline. Bias arises as subjects that are assigned to a treatment group due to information e.g., 12 h after baseline can—by definition—not have experienced the outcome until that time.
Stabilized weights	IPTW/IPCW weights with the marginal or conditional probability of treatment/being uncensored in the numerator. For IPCW, this implies the estimation of the effect for a pseudopopulation in which censoring is *random*.
Unstabilized weights	IPTW/IPCW weights with 1 (unity) in the numerator. For IPCW, this implies the estimation of the effect for a pseudopopulation in which censoring is *absent*.
Positivity assumption	Assumption that the probability of receiving each treatment option is larger than zero for all subjects.
Area of common support	Range of probabilities in which the distributions of the treatment probabilities for the different treatment options have overlap.

### 2.2 Target Trial Emulation

We designed a hypothetical trial investigating the effect of initiating vancomycin versus initiating one of several minimally nephrotoxic alternative antibiotics on 14‐day risk of AKI in adult non‐dialysis dependent AKI‐free ICU admissions. We provide a brief description of the trial and emulation protocol and refer to the supplements for more information (Supplementary Data S3).

### 2.3 Eligibility Criteria

The eligibility criteria in the hypothetical trial were defined as follows: adult, non‐dialysis dependent AKI‐free ICU admissions with a suspected bacterial infection that was perceived to be treatable with vancomycin or one of the six following alternative antibiotics: clindamycin, linezolid, teicoplanin, meropenem, cefazolin, or daptomycin. Additionally, ICU admissions were only eligible for enrollment after 24 h and before 7 days post ICU admission. Lastly, a baseline serum creatinine (SCr) measurement in the first 24 h of the ICU admission was required for inclusion. Our emulation used identical criteria except that, due to the lack of registered indications linked to drug administrations in the structured EHR data in The Netherlands, we assumed that initiation of one of the study antibiotics reflected a suspected bacterial infection that was perceived to be treatable with the initiated antibiotic.

### 2.4 Treatment Strategies and Assignment

We compared the initiation of vancomycin to the initiation of one of the six above‐mentioned alternative antibiotics, with the initiation timepoint constituting the index timepoint (time zero). Given our aim to investigate the nephrotoxicity of vancomycin, we explicitly added to the treatment strategies to not initiate the other treatment strategy after initiation. The alternative antibiotics were selected based on two factors. First, their overlapping indications with vancomycin according to Dutch antibiotics guidelines and the drug knowledge database *Informatorium Medicamentorum* (IM) of the Royal Dutch Pharmacists Association [27, 28]. Second, minimal nephrotoxicity according to the IM and a recent consensus on the nephrotoxicity of drugs [27, 29]. In the hypothetical trial, the strategies were assigned randomly and non‐blinded when eligibility criteria were met. In the emulation, ICU admissions were assigned to the treatment strategies according to the drug administration data. Admissions with simultaneous initiation (i.e., within the same minute) of vancomycin and an alternative were excluded.

### 2.5 Follow‐Up

In the hypothetical trial, follow‐up started at the time of treatment assignment and ended at AKI diagnosis, loss to follow‐up, initiation of the other treatment strategy, initiation of kidney replacement therapy (KRT, without preceding AKI), death, or 14 days after treatment assignment [30], whichever occurred first. In our emulation, follow‐up started at initiation of treatment and ended at AKI diagnosis, discharge from the ICU, initiation of the other treatment strategy, initiation of KRT (without preceding AKI), death, or 14 days after treatment initiation, whichever occurred first.

### 2.6 Outcome

The outcome of interest was AKI according to the ‘Kidney Disease: Improving Global Outcomes’ (KDIGO) 2012 criteria [31]. In our emulation, we applied the SCr AKI criteria and used the first SCr in the first 24 h of the ICU admission as the SCr baseline.

### 2.7 Statistical Analysis

Baseline characteristics of the ICU admissions were described through counts with percentages or medians with interquartile ranges as appropriate.

We aimed to estimate a per protocol effect. ICU admissions were censored at initiation of the other treatment strategy, 14 days after treatment initiation and ICU discharge. Weighted cumulative incidence curves were estimated for each treatment strategy using the Aalen‐Johansen method while accounting for two competing risks: initiation of KRT (without a preceding AKI) and death. The weights served two goals: adjustment for confounding through inverse probability of treatment weighting (IPTW) and adjustment for selection bias through inverse probability of censoring weighting (IPCW). We thereby aimed to estimate the causal effect for a pseudopopulation in which the treatment strategy assignment was independent of potential confounders and censoring was random.

We calculated the probability of initiating each treatment strategy for every ICU admission through a logistic regression model with the treatment strategy as the dependent variable and a set of potential confounders as independent variables. The potential confounders were identified a priori based on the AKI literature [18, 31, 32, 33, 34, 35] and expert consensus (Supplementary Data S4), in line with the confounder selection literature [36, 37, 38, 39]. We included potential nephrotoxic drugs in the ICU according to the recent consensus and side‐effects listed in the IM (Supplementary Data S5) [27, 29]. Drugs with at least a ‘possible to probable’ nephrotoxic potential in the consensus or a renal side‐effect frequency of at least 1% in the IM were added. Using the obtained treatment probabilities, we calculated stabilized weights to be applied in IPTW.

For IPCW we calculated the daily time‐varying probability of remaining uncensored for each ICU admission through a pooled logistic regression model. These probabilities were subsequently used to calculate time‐varying stabilized weights. We identified a set of independent variables for the pooled logistic regression model that largely overlapped with the variables in the treatment probability model, but additionally contained longitudinal versions to model the time‐varying probability of remaining uncensored.

We visually inspected the overlap in the predicted treatment probability distributions between the different treatment strategies to detect potential positivity violations, and assessed the balance of the potential confounders across the treatment strategies after IPTW to detect potential residual confounding. We considered absolute standardized mean differences lower than or equal to 0.1 and variance ratios between 0.5 and 2 well balanced [40, 41, 42]. Splines and interaction terms were added to the treatment probability model to optimize balance where needed [43]. We additionally assessed whether the mean of the IPCW weights remained approximately one during follow‐up, as this is a necessary condition for correct model specification [44]. To obtain the final weights, we multiplied the IPTW weights by the IPCW weights. All weights were truncated to the 1st and 99th percentiles.

Given the time lag of 24–48 h between changes in renal function and SCr, any effect of vancomycin on AKI incidence would only become apparent after 24–48 h post initiation [8, 45]. We therefore calculated absolute risks and risk differences at both 2 and 14 days after initiation to summarize our effect estimate.

We conducted five sensitivity analyses. The first two investigated the impact of excluding the admissions outside the area of common support and not truncating the weights. As part of the estimated total effect of vancomycin on AKI might be mediated by competing events (e.g., death), we conducted a third sensitivity analysis in which we estimated the effect not mediated by competing events (by considering the competing events as censoring events and obtaining the weighted [IPTW and IPCW] cumulative incidence curves using the Kaplan–Meier method [46]). We additionally investigated the sensitivity of our estimates to the missing data imputation approach by applying multiple imputation by chained equations (using predictive mean matching to obtain five imputed datasets). Lastly, we investigated the impact of IPCW on the estimates for two censoring events separately: initiation of the other treatment strategy and discharge from the ICU.

### 2.8 Exploratory Dose–Response Analysis

We conducted an exploratory analysis to investigate a potential dose–response relationship between vancomycin and the 14‐day risk of AKI. We calculated the total received vancomycin dose in mg/kg in the first 24 h of therapy for the ICU admissions that initiated this antibiotic and created two groups: a lower starting dose (≤ 35 mg/kg) and a higher starting dose (> 35 mg/kg). We subsequently repeated our procedure to calculate adjusted absolute risks and risk differences at 2 and 14 days after initiation and compared both the lower and higher vancomycin starting doses to the initiation of an alternative antibiotic.

Importantly, as this exploratory analysis was susceptible to immortal time bias due to the assignment of treatment groups based on post‐baseline information, we utilized a landmark approach by excluding admissions that were lost to follow‐up in the first 24 h after initiation and started follow‐up after this day [47]. More information about the exploratory analysis is provided in Supplementary Data S2.

We applied cluster bootstrapping with 1000 replications to obtain 95% confidence intervals (CIs) for all estimates. The analyses were conducted in R version 4.3.2 [48].

## 3 Results

### 3.1 Eligible ICU Admissions

**FIGURE 2:**
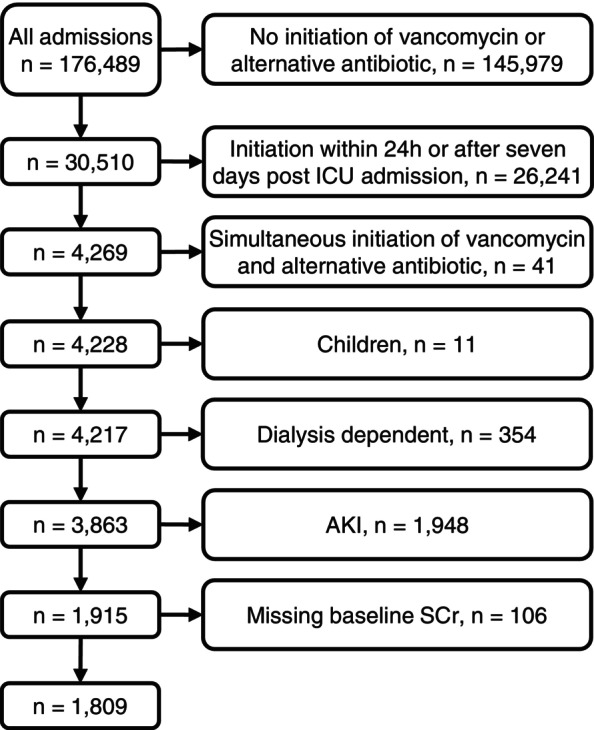
Description of the number of included ICU admissions after application of the eligibility and exclusion criteria.

Our entire cohort consisted of 176 489 ICU admissions of whom 30 510 initiated one of the treatment strategies and 1 809 met all eligibility criteria (Figure 2). Of these, 887 initiated vancomycin and 922 initiated an alternative antibiotic. Baseline characteristics and crude outcomes for the ICU admissions in the two treatment groups are presented in Table 2 (see Supplementary Data S6 and S7 for an extended version and information on missing data).

**TABLE 2:** Baseline characteristics and crude outcomes—primary analysis.

Characteristic	Alternative (*n* = 922)	Vancomycin (*n* = 887)
Age (years), median (Q1–Q3)	63.0 (52.0–72.0)	65.0 (54.0–73.0)
Male sex, no. (%)	555 (60.2)	549 (61.9)
Admission type		
Medical, no. (%)	677 (73.5)	551 (62.1)
Emergency surgical, no. (%)	151 (16.4)	191 (21.5)
Elective surgical, no. (%)	93 (10.1)	145 (16.3)
APACHE IV score, median (Q1–Q3)	67.0 (48.0–83.0)	69.0 (56.0–85.5)
SCr baseline (mg/dL), median (Q1–Q3)	0.9 (0.7–1.2)	0.9 (0.7–1.2)
Acute AKI risk factors		
Acute heart failure, no. (%)	102 (11.1)	117 (13.2)
Graft or transplant surgery, no. (%)	46 (5.0)	71 (8.0)
Hypoalbuminemia, no. (%)	777 (84.3)	782 (88.2)
Hypotension, no. (%)	310 (33.6)	341 (38.4)
Hypovolemia, no. (%)	23 (2.5)	25 (2.8)
Major surgery, no. (%)	209 (22.7)	269 (30.3)
Mechanical ventilation, no. (%)	586 (63.6)	668 (75.3)
Sepsis–admission diagnosis, no. (%)	141 (15.3)	151 (17.0)
Sepsis–longitudinal, no. (%)	326 (35.4)	374 (42.2)
Trauma, no. (%)	96 (10.4)	25 (2.8)
Chronic AKI risk factors		
Alcohol abuse, no. (%)	193 (20.9)	214 (24.1)
Cardiovascular disease, no. (%)	272 (29.5)	221 (24.9)
Chronic kidney disease, no. (%)	12 (1.3)	24 (2.7)
Chronic pulmonary disease, no. (%)	159 (17.2)	118 (13.3)
Diabetes mellitus, no. (%)	138 (15.0)	136 (15.3)
Liver disease, no. (%)	12 (1.3)	13 (1.5)
Obesity, no. (%)	128 (13.9)	140 (15.8)
Nephrotoxin exposure		
C03CA01–furosemide, no. (%)	336 (36.4)	482 (54.3)
J01EE01–sulfamethoxazole and trimethoprim, no. (%)	38 (4.1)	57 (6.4)
J01GB03–gentamicin, no. (%)	140 (15.2)	149 (16.8)
J02AA01–amphotericin B, no. (%)	9 (1.0)	27 (3.0)
M01AB05–diclofenac, no. (%)	25 (2.7)	11 (1.2)
AKI, no. (%)	125 (13.6)	167 (18.8)
AKI stage		
Stage 1, no. (%)	85 (68.0)	106 (63.5)
Stage 2, no. (%)	14 (11.2)	27 (16.2)
Stage 3, no. (%)	26 (20.8)	34 (20.4)
KRT, no. (%)	22 (17.6)	23 (13.8)

### 3.2 Effect Estimates

The treatment and censoring models were fitted with 48 and 74 potential confounding variables, respectively (Supplementary Data S8). The distributions of the calculated treatment initiation probabilities across the different treatment strategies showed sufficient overlap, and the potential confounders were well balanced after IPTW (Figures S9.1 and S9.2). The mean of the IPCW weights remained approximately one during follow‐up (Figure S10.1).

**FIGURE 3:**
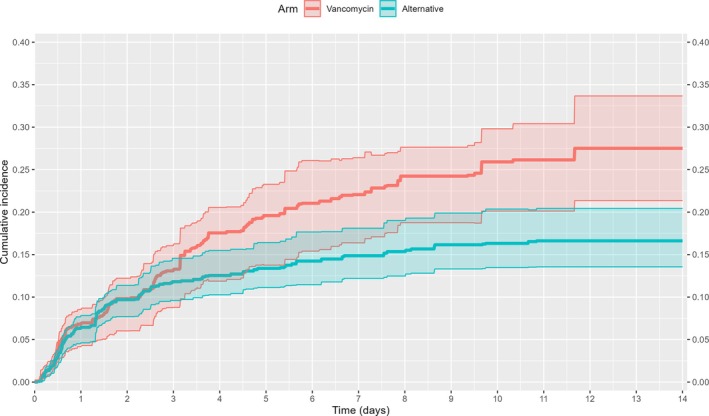
Adjusted (IPTW and IPCW) cumulative incidence curves with pointwise 95% CIs, stratified by treatment.

After adjustment, initiation of vancomycin was associated with a higher risk of AKI at 14 days of follow‐up compared to initiation of an alternative antibiotic (0.28 [95% CI 0.21–0.34] vs. 0.17 [95% CI 0.14–0.20]; risk difference 0.11 [95% CI 0.04–0.19]), but not at 2 days of follow‐up (0.10 [95% CI 0.06–0.12] vs. 0.10 [95% CI 0.08–0.11]; risk difference 0.00 [95% CI −0.03–0.03], Table 3, Figure 3, Supplementary Data S11). The adjusted cumulative incidence curves indicated that the increased risk of AKI associated with vancomycin became apparent early in the follow‐up period between 2 and 14 days (Figure 3).

**TABLE 3:** Unadjusted and adjusted (IPTW and IPCW) estimates with 95% CIs for the primary analysis.

	Day	Risk vancomcin	Risk alternative	Risk difference
Unadjusted	2	0.11 (0.07–0.14)	0.10 (0.08–0.12)	0.01 (−0.02–0.04)
Adjusted	2	0.10 (0.06–0.12)	0.10 (0.08–0.11)	0.00 (−0.03–0.03)
Unadjusted	14	0.29 (0.23–0.33)	0.24 (0.20–0.29)	0.05 (−0.03–0.10)
Adjusted	14	0.28 (0.21–0.34)	0.17 (0.14–0.20)	0.11 (0.04–0.19)

The sensitivity analyses resulted in similar estimates, although not truncating the weights produced a bigger 14‐day risk difference point estimate (0.15 [95% CI 0.05–0.22]). While IPCW for initiation of the other treatment strategy had a small impact, IPCW for discharge had a large impact on the estimates. The effect not mediated by competing events was comparable to the total effect estimated in the main analysis (Supplementary Data S12).

### 3.3 Exploratory Dose–Response Analysis

After excluding the ICU admissions that were lost to follow‐up before the 24‐h landmark, 1328 admissions remained, of whom 652 initiated an alternative antibiotic, 322 initiated vancomycin with the lower starting dose, and 354 initiated vancomycin with the higher starting dose. Baseline characteristics are presented in Supplementary Data S13 and S14. The distributions of the treatment initiation probabilities again showed sufficient overlap (Figure S15.1). While the potential confounders were generally well‐balanced after IPTW, one variable (encoding the presence of admissions in one specific ICU) remained slightly imbalanced after the modeling procedure (Figures S15.2 and S15.3). The mean of the IPCW weights again remained approximately one during follow‐up (Figure S16.1).

After adjustment, both the higher and the lower vancomycin doses were associated with an increased risk of AKI at 14 days of follow‐up compared to the initiation of an alternative antibiotic, but the higher vancomycin dose had a bigger risk difference point estimate (0.17 [95% CI 0.05–0.25] vs. 0.10 [95% CI 0.01–0.19], Table 4, Supplementary Data S17).

**TABLE 4:** Unadjusted and adjusted (IPTW and IPCW) estimates with 95% CIs for the exploratory analysis.

Analysis	Day	Risk vancomycin higher dose	Risk vancomycin lower dose	Risk alternative	Risk difference vancomycin higher dose versus alternative	Risk difference vancomycin lower dose versus alternative
Unadjusted	2	0.04 (0.02–0.06)	0.05 (0.03–0.07)	0.03 (0.02–0.05)	0.01 (−0.01–0.03)	0.02 (−0.01–0.03)
Adjusted	2	0.04 (0.01–0.07)	0.03 (0.01–0.05)	0.04 (0.02–0.06)	0.00 (−0.03–0.02)	−0.01 (−0.03–0.02)
Unadjusted	14	0.22 (0.17–0.25)	0.27 (0.20–0.32)	0.19 (0.14–0.24)	0.03 (−0.05–0.09)	0.08 (0.00–0.14)
Adjusted	14	0.30 (0.19–0.37)	0.23 (0.15–0.32)	0.13 (0.10–0.16)	0.17 (0.05–0.25)	0.10 (0.01–0.19)

The sensitivity analyses again resulted in similar estimates and consistently indicated a bigger 14‐day risk difference point estimate of the higher vancomycin dose compared to the lower vancomycin dose (Supporting Information S18).

## 4 Discussion

To the best of our knowledge, this is the first TTE study investigating the effect of initiating vancomycin versus initiating one of several minimally nephrotoxic alternative antibiotics on the 14‐day risk of AKI in adult ICU admissions. We found that the initiation of vancomycin is associated with a higher risk of AKI compared to the initiation of the alternative antibiotics, but this effect only became apparent after 2 days post initiation in line with the time lag between changes in renal function and SCr.

It is difficult to compare our effect estimate to the results of previous research, such as the hazard ratios (HRs) of 1.24 and 1.55 in two recent observational studies with large cohorts of ICU patients [16, 18], and the RR of 2.45 in a meta‐analysis of RCTs (hospitalized patients) [10]. First, the study populations are likely different, and thereby the distributions of potential effect‐modifying patient characteristics (e.g., chronic kidney disease and age). Second, estimates were often presented through relative measures, such as the HR or RR. The HR generally has no causal interpretation due to built‐in selection bias; the hazard function conditions on the subjects still at risk, and this status can be affected by both the treatment and unmeasured causes of the outcome [46, 49, 50]. Furthermore, summarizing a time‐varying effect in a single HR may inaccurately characterize a delayed effect [51]. RRs do not suffer from the built‐in selection bias but are still sensitive to the time lag between AKI and the utilized AKI marker (which likely varies between markers, such as SCr and urine output [UO]). An equal incidence of AKI added to each group under comparison during the first few days of follow‐up (resulting from injuries before baseline) will alter the RR unless there is no effect. A quantification of the effect through cumulative incidence curves along with absolute risks and risk differences does not suffer from the above limitations. Furthermore, absolute risks seem to be preferred by both patients and investigators over relative measures [52]. Increased adoption of the absolute measures in future studies could therefore improve the validity and comparability of effect estimates.

The time lag between changes in renal function and SCr does present an opportunity to further assess estimates for residual confounding, because the AKI incidence in the first few days of follow‐up could serve as a negative control outcome [53]. There cannot be an effect of the treatment initiation on the SCr‐based AKI incidence in the first few days of follow‐up, because those AKIs happened before initiation. An effect in the first few days would indicate residual confounding; however, we did not find such an effect.

The higher risk of AKI associated with vancomycin became apparent early in the period between 2 and 14 days of follow‐up according to the adjusted cumulative incidence curves. Given that an SCr measured 2 days post initiation roughly reflects renal function at or shortly after initiation, our results may indicate that even short courses of vancomycin of one or 2 days are associated with a higher risk of AKI, and that a potential necessary induction period may be short (i.e., one or 2 days). Put another way, if longer courses or a longer induction period would be required (e.g., of 4 days), we would expect the effect to become apparent only later during follow‐up (e.g., after day six).

We additionally present an important exploratory investigation of a potential dose–response relationship through the usage of drug administration data. This additional line of inquiry next to the investigation of vancomycin trough concentrations is important, for the latter approach may be at risk of reverse‐causation bias [8, 9, 10, 14]. The results of our exploratory analysis should, however, be interpreted with caution, as the dosage groups were data‐driven and may have limited clinical relevance. Furthermore, although the higher dose had a higher risk difference point estimate than the lower dose, the overlapping CIs indicated that the absence of a dose–response relationship was also compatible with the data.

## 5 Strengths and Limitations

This study has several strengths. First, we utilized the TTE framework to design a hypothetical trial comparing vancomycin with an active comparator control group and emulated it using a large observational cohort with admissions to 15 ICUs. Second, we adjusted for potential confounders identified through existing knowledge and additionally adjusted for potential selection bias. Third, we quantified the effect through absolute risks and risk differences at both 2 and 14 days of follow‐up, which is important given the known time lag [8, 54, 55, 56].

Our approach also has limitations. First, although we used an active comparator design, the specific treatment indications may not have been fully exchangeable between the vancomycin and alternative antibiotics groups, and the alternative antibiotics were likely not individually interchangeable. While this may have led to violations of the positivity assumption and residual confounding, we did not detect this in our treatment probability overlap and balance assessments and deem this risk as low. Although we did note a slight covariate imbalance for one variable in our *exploratory* dose–response analysis (encoding the presence of admissions in one specific ICU), we deem the magnitude of residual confounding as small given the minor imbalance combined with the low number of admissions in this ICU (about 2% of all admissions in the exploratory analysis). Second, due to data sharing restrictions in The Netherlands, we did not have pre‐ICU medication data, which hindered the adjustment for pre‐admission drug exposure and the identification of new users. To mitigate this limitation, we used a washout period by excluding ICU admissions that received vancomycin or an alternative antibiotic in the first 24 h of the ICU admission. Third, we did not apply the KDIGO AKI UO criteria for the diagnosis of AKI during follow‐up due to highly incomplete and heterogeneous UO data, which is a common issue in studies utilizing routinely collected data [56, 57]. SCr therefore remains the primary marker for AKI in studies using such data [56]. Lastly, causal inference using observational data requires strong assumptions, including conditional exchangeability and positivity for both IPTW and IPCW. Altering our observational study designs or data collection to allow for weaker assumptions could increase the strength of the evidence. For example, discharge from the ICU was a censoring event in our study as no outcome information was available after ICU discharge, and we adjusted for selection bias through IPCW. If follow‐up studies can collect reliable outcome information after ICU discharge, these assumptions may be somewhat relaxed.

## 6 Clinical Implications and Future Research

Our findings underline the importance of clinical vancomycin‐induced AKI prevention strategies. These include dosing vancomycin according to renal function, therapeutic drug monitoring, and the consideration of a non‐nephrotoxic alternative antibiotic if possible [58, 59]. Future research may address the limitations of our TTE approach and thereby increase the strength of the evidence. This could, for example, encompass the pairwise comparison with multiple alternative antibiotics, the adjustment for pre‐ICU drug exposure, or additional utilization of the UO AKI criteria if the UO data is of sufficient quality.

## 7 Conclusions

Our findings indicate that vancomycin causes a higher risk of AKI compared to the investigated alternative antibiotics. We recommend clinicians to be compliant with clinical vancomycin‐induced AKI prevention strategies. Future research on dose–response relationships is warranted.

### 7.1 Plain Language Summary

The potential of vancomycin to cause acute kidney injury (AKI) in patients admitted to the intensive care unit (ICU) is subject to debate due to suboptimal designs of past studies. To provide stronger evidence, we used the target trial emulation framework to design and emulate a hypothetical randomized controlled trial using a large observational dataset with routinely collected data of 15 Dutch ICUs. We aimed to estimate the effect of initiating vancomycin versus one of several alternative antibiotics on the 14‐day risk of AKI. In our statistical analysis, we adjusted for both potential confounding and selection bias. We included 1809 ICU admissions. After adjustment, vancomycin was associated with a higher 14‐day risk of AKI compared to the alternative antibiotics (0.28 [95% confidence interval (CI) 0.21–0.34] vs. 0.17 [95% CI 0.14–0.20]; risk difference 0.11 [95% CI 0.04–0.19]). Our findings indicate that vancomycin causes a higher risk of AKI compared to the alternative antibiotics. We recommend clinicians to be compliant with vancomycin‐induced AKI prevention strategies, such as therapeutic drug monitoring or the consideration of an alternative antibiotic if possible.

## Author Contributions

Concept and design: Izak A.R. Yasrebi‐de Kom, Joanna E. Klopotowska, Giovanni Cinà, Vianda S. Stel, Nicholas C. Chesnaye, Dave A. Dongelmans, Kitty J. Jager, Ameen Abu‐Hanna, and Nicolette F. de Keizer. Acquisition of the data: Joanna E. Klopotowska, Nicolette F. de Keizer, Dylan W. de Lange, and Dave A. Dongelmans. Data pre‐processing and understanding: Izak A.R. Yasrebi‐de Kom, Joanna E. Klopotowska, Dylan W. de Lange, and Dave A. Dongelmans. Analysis: Izak A.R. Yasrebi‐de Kom, Nicholas C. Chesnaye, and Giovanni Cinà. Interpretation: All authors. Drafting the manuscript: Izak A.R. Yasrebi‐de Kom. Critically revising the manuscript: All authors. Funding: Joanna E. Klopotowska, Ameen Abu‐Hanna, Nicolette F. de Keizer, Dave A. Dongelmans, and Dylan W. de Lange. All authors approved the submitted manuscript.

## Ethics Statement

This study was exempted from requiring ethics approval (waiver W19_433 # 19.499) on November 14th 2019 by the Medical Ethics Committee of the Amsterdam University Medical Centers, location University of Amsterdam.

## Consent

Patient informed consent was waived.

## Conflicts of Interest

For the duration of this research, Giovanni Cinà has held a partial appointment at the company Pacmed, as well as stock appreciation options. The other authors declare no conflicts of interest.

## Supporting information


**Supplementary Data S1:** pds70205‐sup‐0001‐Supinfo.

## Data Availability

The datasets used in this study are not publicly available due to the data sharing agreements with the participating ICUs. Access to the data might only be provided after explicit consent from each separate participating ICU.

## Appendix A

The RESCUE Study Group Members:

C. S. C. Bouman (Amsterdam University Medical Center, Department of Intensive Care Medicine, Amsterdam, The Netherlands), E. N. van Roon (Department of Clinical Pharmacy and Pharmacology, Medical Center Leeuwarden, Leeuwarden, The Netherlands), J. ten Cate (Department of Intensive Care, The Netherlands Cancer Institute, Amsterdam, The Netherlands), P. F. Schutte (Department of Intensive Care, The Netherlands Cancer Institute, Amsterdam, The Netherlands), D. van Balen (Department of Pharmacy & Pharmacology, The Netherlands Cancer Institute, Amsterdam, The Netherlands), S. Hendriks (Department of Intensive Care, Albert Schweitzer Ziekenhuis, Dordrecht, The Netherlands), C. Lau (Department of Hospital Pharmacy, Albert Schweitzer Ziekenhuis, Dordrecht, The Netherlands), W. J. Vermeijden (Department of Intensive Care, Medisch Spectrum Twente, Enschede, The Netherlands), A. Beishuizen (Department of Intensive Care, Medisch Spectrum Twente, Enschede, The Netherlands), J. B. Masselink (Department of Clinical Pharmacy, Medisch Spectrum Twente, Enschede, The Netherlands), P. E. Spronk (Department of Intensive Care Medicine, Gelre Hospitals, Apeldoorn, The Netherlands), H. J. M. van Kan (Department of Clinical Pharmacy, Gelre Hospitals, Apeldoorn, The Netherlands), P. W. de Feiter (Department of Intensive Care, Franciscus Gasthuis & Vlietland, Rotterdam, The Netherlands, current affiliation: Amsterdam University Medical Center, Department of Intensive Care Medicine, Amsterdam, The Netherlands), E.‐J. Wils (Department of Intensive Care, Franciscus Gasthuis & Vlietland, Rotterdam, The Netherlands), A. Wieringa (Department of Clinical Pharmacy, Isala Hospital, Zwolle, The Netherlands), A. J. Valkenburg (Department of Intensive Care, Isala Hospital, Zwolle, The Netherlands), W. M. van den Bergh (Department of Critical Care, University Medical Center Groningen, University of Groningen, Groningen, The Netherlands), M. H. Renes (Department of Critical Care, University Medical Center Groningen, University of Groningen, Groningen, The Netherlands), W. Bult (Department of Critical Care, University Medical Center Groningen, University of Groningen, Groningen, The Netherlands, Department of Clinical Pharmacy and Pharmacology, University Medical Center Groningen, University of Groningen, Groningen, The Netherlands), E. de Jonge (Department of Intensive Care, Leiden University Medical Center, Leiden, The Netherlands), M. Hoeksema (Department of Anesthesiology, Intensive Care and Painmanagement, Zaans Medisch Centrum, Zaandam, The Netherlands), E. J. Wesselink (Department of Clinical Pharmacy, Zaans Medisch Centrum, Zaandam, The Netherlands), I. M. Purmer (Department of Intensive Care, Haga Hospital, The Hague, The Netherlands), B. E. Bosma (Department of Hospital Pharmacy, Haga Hospital, The Hague, The Netherlands), S. H. W. van Bree (Department of Intensive Care, Ziekenhuis Gelderse Vallei, Ede, The Netherlands), H. J. W. Lammers (Department of Hospital Pharmacy, Ziekenhuis Gelderse Vallei, Ede, The Netherlands), R. J. Bosman (Department of Intensive Care, Onze Lieve Vrouwe Gasthuis, Amsterdam, The Netherlands), E. J. F. Franssen (Department of Clinical Pharmacy, Onze Lieve Vrouwe Gasthuis, Amsterdam, The Netherlands), A. Karakus (Department of Intensive Care, Diakonessenhuis Utrecht, Utrecht, The Netherlands), M. Sigtermans (Department of Intensive Care, Diakonessenhuis Utrecht, Utrecht, The Netherlands) and E. M. Kuck (Department of Hospital Pharmacy, Diakonessenhuis Utrecht, Utrecht, The Netherlands).
