# Supplementary material for: Vancomycin‐Induced Acute Kidney Injury in Intensive Care Patients: A Target Trial Emulation Study Using Multicenter Routinely Collected Data
Source: Pharmacoepidemiol Drug Saf. 2025 Aug 28;34(9):e70205. doi: 10.1002/pds.70205 (PMC12394726; doi:10.1002/pds.70205)
Supplement: Supplementary file 1 — Supplementary Data S1: pds70205‐sup‐0001‐Supinfo. [file PDS-34-e70205-s001.pdf]

Vancomycin-induced acute kidney injury in intensive care patients: a target trial emulation study using multicenter routinely collected data

Izak A.R. Yasrebi-de Kom[1,2]*, Kitty J. Jager[1,2], Vianda S. Stel[1,2], Nicholas C. Chesnaye[3,2], Ameen Abu-Hanna[1,2], Nicolette F. de Keizer[1,2], Dylan W. de Lange[4], Dave A. Dongelmans[5,2], Joanna E. Klopotowska[1,2]**, Giovanni Cinà[1,2,6]**, on behalf of the RESCUE Study Group***

[1]Amsterdam University Medical Center, Department of Medical Informatics, Amsterdam, The Netherlands
[2]Amsterdam Public Health, Amsterdam, The Netherlands
[3]Amsterdam University Medical Center, ERA Registry, Department of Medical Informatics, Amsterdam, The Netherlands
[4]University Medical Center Utrecht, Department of Intensive Care and Dutch Poison Information Center, Utrecht, The Netherlands
[5]Amsterdam University Medical Center, Department of Intensive Care Medicine, Amsterdam, The Netherlands
[6]University of Amsterdam, Institute for Logic, Language and Computation, Amsterdam, The Netherlands

*Correspondence: i.a.r.dekom@amsterdamumc.nl ORCID: 0000-0002-8655-2521
Address: Meibergdreef 15, 1105AZ, Amsterdam, The Netherlands
**Contributed equally to the study
***The RESCUE Study Group Members:

C.S.C. Bouman[5], E.N. van Roon[7], J. ten Cate[8], P.F. Schutte[8], D. van Balen[9], S. Hendriks[10], C. Lau[11], W.J. Vermeijden[12], A. Beishuizen[12], J.B. Masselink[13], P.E. Spronk[14], H.J.M. van Kan[15], P.W. de Feiter[16,5,a], E.-J. Wils[16], A. Wieringa[17], A.J. Valkenburg[18], W.M. van den Bergh[19], M.H. Renes[19], W. Bult[19,20], E. de Jonge[21], M. Hoeksema[22], E.J. Wesselink[23], I.M. Purmer[24], B.E. Bosma[25], S.H.W. van Bree[26], H.J.W. Lammers[27], R.J. Bosman[28], E.J.F. Franssen[29], A. Karakus[30], M. Sigtermans[30] and E.M. Kuck[31]

[7]Department of Clinical Pharmacy and Pharmacology, Medical Center Leeuwarden, Leeuwarden, The Netherlands
[8]Department of Intensive Care, The Netherlands Cancer Institute, Amsterdam, The Netherlands
[9]Department of Pharmacy & Pharmacology, The Netherlands Cancer Institute, Amsterdam, The Netherlands
[10]Department of Intensive Care, Albert Schweitzer Ziekenhuis, Dordrecht, The Netherlands
[11]Department of Hospital Pharmacy, Albert Schweitzer Ziekenhuis, Dordrecht, The Netherlands
[12]Department of Intensive Care, Medisch Spectrum Twente, Enschede, The Netherlands
[13]Department of Clinical Pharmacy, Medisch Spectrum Twente, Enschede, The Netherlands
[14]Department of Intensive Care Medicine, Gelre Hospitals, Apeldoorn, The Netherlands
[15]Department of Clinical Pharmacy, Gelre Hospitals, Apeldoorn, The Netherlands
[16]Department of Intensive Care, Franciscus Gasthuis & Vlietland, Rotterdam, The Netherlands
[17]Department of Clinical Pharmacy, Isala Hospital, Zwolle, The Netherlands
[18]Department of Intensive Care, Isala Hospital, Zwolle, The Netherlands
[19]Department of Critical Care, University Medical Center Groningen, University of Groningen, Groningen, The Netherlands
[20]Department of Clinical Pharmacy and Pharmacology, University Medical Center Groningen, University of Groningen, Groningen, The Netherlands
[21]Department of Intensive Care, Leiden University Medical Center, Leiden, The Netherlands
[22]Department of Anesthesiology, Intensive Care and Painmanagement, Zaans Medisch Centrum, Zaandam, The Netherlands
[23]Department of Clinical Pharmacy, Zaans Medisch Centrum, Zaandam, The Netherlands
[24]Department of Intensive Care, Haga Hospital, The Hague, The Netherlands
[25]Department of Hospital Pharmacy, Haga Hospital, The Hague, The Netherlands
[26]Department of Intensive Care, Ziekenhuis Gelderse Vallei, Ede, The Netherlands
[27]Department of Hospital Pharmacy, Ziekenhuis Gelderse Vallei, Ede, The Netherlands
[28]Department of Intensive Care, Onze Lieve Vrouwe Gasthuis, Amsterdam, The Netherlands
[29]Department of Clinical Pharmacy, Onze Lieve Vrouwe Gasthuis, Amsterdam, The Netherlands
[30]Department of Intensive Care, Diakonessenhuis Utrecht, Utrecht, The Netherlands
[31]Department of Hospital Pharmacy, Diakonessenhuis Utrecht, Utrecht, The Netherlands
[a]Currently affiliated with the secondary affiliation

Supplements – Table of contents

Supplement S1. RECORD-PE checklist of items ---------- Page 3
Supplement S2. Supplementary methods ---------- Page 7
Supplement S3. Target trial emulation protocol ---------- Page 9
Supplement S4. Potential confounders ---------- Page 11
Supplement S5. Potential nephrotoxic drugs ---------- Page 13
Supplement S6. Extended baseline characteristics and crude outcomes – primary analysis ---------- Page 15
Supplement S7. Extended baseline characteristics and crude outcomes with missing data – primary analysis ---------- Page 20
Supplement S8. Selected variables ---------- Page 26
Supplement S9. Overlap and balance assessment for IPTW – primary analysis ---------- Page 29
Supplement S10. IPCW weights – primary analysis ---------- Page 31
Supplement S11. Estimates – primary analysis ---------- Page 32
Supplement S12. Sensitivity analyses – primary analysis ---------- Page 33

Supplement S13. Extended baseline characteristics and crude outcomes – exploratory analysis ---------- Page 34
Supplement S14. Extended baseline characteristics and crude outcomes with missing data – exploratory analysis ---------- Page 37
Supplement S15. Overlap and balance assessments for IPTW – exploratory analysis ---------- Page 41
Supplement S16. IPCW weights – exploratory analysis ---------- Page 44
Supplement S17. Estimates – exploratory analysis ---------- Page 45
Supplement S18. Sensitivity analyses – exploratory analysis ---------- Page 46

Back to table of contents

Supplement S1. RECORD-PE checklist of items

The RECORD statement for pharmacoepidemiology (RECORD-PE) checklist of items, extended from the STROBE and RECORD statements, which should be reported in non-interventional pharmacoepidemiological studies using routinely collected health data

Item No	STROBE items	RECORD items	RECORD-PE items	Page No
**Title and abstract**				
1	(a) Indicate the study's design with a commonly used term in the title or the abstract.<br>(b) Provide in the abstract an informative and balanced summary of what was done and what was found.	1.1: The type of data used should be specified in the title or abstract. When possible, the name of the databases used should be included.<br>1.2: If applicable, the geographical region and timeframe within which the study took place should be reported in the title or abstract.<br>1.3: If linkage between databases was conducted for the study, this should be clearly stated in the title or abstract.	—	1-4
**Introduction**				
Background rationale				
2	Explain the scientific background and rationale for the investigation being reported.	—	—	5
Objectives				
3	State specific objectives, including any prespecified hypotheses.	—	—	6
**Methods**				
Study design				
4	Present key elements of study design early in the paper.	—	4.a: Include details of the specific study design (and its features) and report the use of multiple designs if used.<br>4.b: The use of a diagram(s) is recommended to illustrate key aspects of the study design(s), including exposure, washout, lag and observation periods, and covariate definitions as relevant.	6
Setting				
5	Describe the setting, locations, and relevant dates, including periods of recruitment, exposure, follow-up, and data collection.	—	—	6-7
Participants				
6	(a) Cohort study—give the eligibility criteria, and the sources and methods of selection of participants. Describe methods of follow-up. Case-control study—give the eligibility criteria, and the sources and methods of case ascertainment and control selection. Give the rationale for the choice of cases and controls. Cross sectional study—give the eligibility criteria, and the sources and methods of selection of participants.	6.1: The methods of study population selection (such as codes or algorithms used to identify participants) should be listed in detail. If this is not possible, an explanation should be provided.<br>6.2: Any validation studies of the codes or algorithms used to select the population should be referenced. If validation was conducted for this study and not published elsewhere, detailed	6.1.a: Describe the study entry criteria and the order in which these criteria were applied to identify the study population. Specify whether only users with a specific indication were included and whether patients were allowed to enter the study population once or if multiple entries were permitted. See explanatory document for guidance related to matched designs.	6-7

Back to table of contents

	(b) Cohort study—for matched studies, give matching criteria and number of exposed and unexposed. Case-control study—for matched studies, give matching criteria and the number of controls per case.	methods and results should be provided.<br>6.3: If the study involved linkage of databases, consider use of a flow diagram or other graphical display to demonstrate the data linkage process, including the number of individuals with linked data at each stage.		
Variables				
7	Clearly define all outcomes, exposures, predictors, potential confounders, and effect modifiers. Give diagnostic criteria, if applicable.	7.1: A complete list of codes and algorithms used to classify exposures, outcomes, confounders, and effect modifiers should be provided. If these cannot be reported, an explanation should be provided.	7.1.a: Describe how the drug exposure definition was developed.<br>7.1.b: Specify the data sources from which drug exposure information for individuals was obtained.<br>7.1.c: Describe the time window(s) during which an individual is considered exposed to the drug(s). The rationale for selecting a particular time window should be provided. The extent of potential left truncation or left censoring should be specified.<br>7.1.d: Justify how events are attributed to current, prior, ever, or cumulative drug exposure.<br>7.1.e: When examining drug dose and risk attribution, describe how current, historical or time on therapy are considered.<br>7.1.f: Use of any comparator groups should be outlined and justified.<br>7.1.g: Outline the approach used to handle individuals with more than one relevant drug exposure during the study period.	6-10
Data sources/measurement				
8	For each variable of interest, give sources of data and details of methods of assessment (measurement). Describe comparability of assessment methods if there is more than one group.	—	8.a: Describe the healthcare system and mechanisms for generating the drug exposure records. Specify the care setting in which the drug(s) of interest was prescribed.	6-9
Bias				
9	Describe any efforts to address potential sources of bias.	—	—	8-10
Study size				
10	Explain how the study size was arrived at.	—	—	6-7
Quantitative variables				
11	Explain how quantitative variables were handled in the analyses. If applicable, describe which groupings were chosen, and why.	—	—	8-9
Statistical methods				
12	(a) Describe all statistical methods, including those used to control for confounding.<br>(b) Describe any methods used to examine subgroups and interactions.	—	12.1.a: Describe the methods used to evaluate whether the assumptions have been met.<br>12.1.b: Describe and justify the use of multiple designs, design features, or analytical approaches.	6-10

Back to table of contents

	(c) Explain how missing data were addressed.<br>(d) Cohort study—if applicable, explain how loss to follow-up was addressed. Case-control study—if applicable, explain how matching of cases and controls was addressed. Cross sectional study—if applicable, describe analytical methods taking account of sampling strategy.<br>(e) Describe any sensitivity analyses.			
Data access and cleaning methods				
12	—	12.1: Authors should describe the extent to which the investigators had access to the database population used to create the study population.<br>12.2: Authors should provide information on the data cleaning methods used in the study.	—	6
Linkage				
12	—	12.3: State whether the study included person level, institutional level, or other data linkage across two or more databases. The methods of linkage and methods of linkage quality evaluation should be provided.	—	6
**Results**				
Participants				
13	(a) Report the numbers of individuals at each stage of the study (eg, numbers potentially eligible, examined for eligibility, confirmed eligible, included in the study, completing follow-up, and analysed).<br>(b) Give reasons for non-participation at each stage.<br>(c) Consider use of a flow diagram.	13.1: Describe in detail the selection of the individuals included in the study (that is, study population selection) including filtering based on data quality, data availability, and linkage. The selection of included individuals can be described in the text or by means of the study flow diagram.	—	10-11
Descriptive data				
14	(a) Give characteristics of study participants (eg, demographic, clinical, social) and information on exposures and potential confounders.<br>(b) Indicate the number of participants with missing data for each variable of interest.<br>(c) Cohort study—summarise follow-up time (eg, average and total amount).	—	—	10-11
Outcome data				
15	Cohort study—report numbers of outcome events or summary measures over time. Case-control study—report numbers in each exposure category, or summary measures of exposure. Cross sectional study—report numbers of outcome events or summary measures.	—	—	10-11
Main results				

Back to table of contents

16	(a) Give unadjusted estimates and, if applicable, confounder adjusted estimates and their precision (eg, 95% confidence intervals). Make clear which confounders were adjusted for and why they were included.<br>(b) Report category boundaries when continuous variables are categorised.<br>(c) If relevant, consider translating estimates of relative risk into absolute risk for a meaningful time period.	—	—	10-12
Other analyses				
17	Report other analyses done—eg, analyses of subgroups and interactions, and sensitivity analyses.	—	—	10-12
**Discussion**				
Key results				
18	Summarise key results with reference to study objectives.	—	—	12
Limitations				
19	Discuss limitations of the study, taking into account sources of potential bias or imprecision. Discuss both direction and magnitude of any potential bias.	19.1: Discuss the implications of using data that were not created or collected to answer the specific research question(s). Include discussion of misclassification bias, unmeasured confounding, missing data, and changing eligibility over time, as they pertain to the study being reported.	19.1.a: Describe the degree to which the chosen database(s) adequately captures the drug exposure(s) of interest.	12-14
Interpretation				
20	Give a cautious overall interpretation of results considering objectives, limitations, multiplicity of analyses, results from similar studies, and other relevant evidence.	—	20.a: Discuss the potential for confounding by indication, contraindication or disease severity or selection bias (healthy adherer/sick stopper) as alternative explanations for the study findings when relevant.	12-15
Generalisability				
21	Discuss the generalisability (external validity) of the study results.	—	—	12-15
**Other information**				
Funding				
22	Give the source of funding and the role of the funders for the present study and, if applicable, for the original study on which the present article is based.	—	—	2
Accessibility of protocol, raw data, and programming code				
22	—	22.1: Authors should provide information on how to access any supplemental information such as the study protocol, raw data, or programming code.	—	6,15

RECORD=reporting of studies conducted using observational routinely collected data; RECORD-PE=RECORD for pharmacoepidemiological research; STROBE=strengthening the reporting of observational studies in epidemiology.

*REFERENCE: Langan SM, Schmidt S, Wing K, Ehrenstein V, Nicholls S, Filion K, Klungel O, Petersen I, Sorensen H, Guttmann A, Harron K, Hemkens L, Moher D, Schneeweiss S, Smeeth L, Sturkenboom M, von Elm E, Wang S, Benchimol EI. The REporting of studies Conducted using Observational Routinely-collected health Data (RECORD) Statement for Pharmacoepidemiology (RECORD-PE). *BMJ* 2018; 363: k3532.

Back to table of contents

Supplement S2. Supplementary methods

This target trial emulation study employed the active comparator, new user design, which amounted to the usage of a control group with subjects that received an alternative antibiotic and the inclusion of subjects that did not receive one of the treatment options before admission to the intensive care unit (ICU) [1].

Data collection

We re-used routinely collected data from electronic health records of admissions to 15 Dutch ICUs between January 2010 and December 2019, containing serum creatinine, serum albumin, leukocyte, core body temperature, systemic drug administration, kidney replacement therapy and arterial blood pressure records. These records were linked to data from the Dutch National Intensive Care Evaluation quality registry (NICE) [2], which included general admission characteristics, demographics, (chronic) comorbidities, physiology measurements, Acute Physiology and Chronic Health Evaluation IV ICU admission diagnoses and scores, sequential organ failure assessment (SOFA) scores and urine output (UO).

The timestamps of all longitudinal data were recorded at minute-level resolution, except the SOFA scores and UO data, which had one value per day. Treatment strategy initiation, outcomes, censoring events and competing events were all recorded at minute-level resolution.

Data processing

Our data contained both static and longitudinal variables. For the longitudinal variables we summarized the data in 24 hour time periods (aligned with the treatment strategy initiation) with clinically relevant functions (Supplement S4). For the baseline variables used in the models we applied a similar approach by summarizing the pre-initiation longitudinal data to single values with identical functions. Although our data contained UO records, the longitudinal UO data after the first 24 hours of the ICU admission was highly heterogeneous across ICUs and often missing. This is a common issue encountered in routinely collected data [3-5]. We therefore did not utilize UO to diagnose AKI during follow-up, but did use it for our eligibility criteria and adjustment for confounding.

Missing value imputation

Missing values in the static data were imputed using the mean or median for continuous or categorical variables, respectively. For missing longitudinal data we applied forward filling. If a value in the first period of the ICU admission was missing, we imputed it using the mean or median for continuous or categorical variables, respectively, and subsequently applied forward filling. Pre-imputation missing value descriptives of the pre-initiation baseline variables are available in Supplements S7 and S14.

Variable selection

We applied a prevalence-based variable selection technique. Selection of variables with a very low prevalence is acceptable as the risk of residual confounding is relatively small in such cases [6]. Variables with binary values and associated prevalences below 1% were dropped. We additionally dropped the pre-initiation maximum serum creatinine variable (from the treatment and censoring models), and the pre-initiation sepsis variable (from the censoring models) as these variables were highly correlated with the serum creatinine baseline and the longitudinal sepsis variables, respectively, resulting in very high variance inflation factors.

Exploratory dose-response analysis

Our data did not allow for the recognition of clinically relevant vancomycin dosage schedules for the first 24 hours of therapy due to the timespan of the data with changing policies (i.e., 2010-2019) and the different dosing options for vancomycin (i.e., continuous and intermittent) with and without a loading dose. We therefore calculated the total received dose of vancomycin in the first 24 hours of therapy and chose a cutoff of 35 mg/kg to create the lower and higher dose groups. This threshold was approximately equal to the mean and median of the distribution of the total dosages in the first 24 hours of therapy, resulting in two groups of roughly equal size.

For IPTW, we applied a multinomial logistic regression model to estimate the probability of receiving each of the three treatment options and calculate stabilized IPTW weights. We compared both the lower and higher vancomycin starting dose to the initiation of an alternative antibiotic.

The calculation of the total vancomycin dose in the first 24 hours of therapy may introduce immortal time bias, as post-baseline drug administration information is used to assign treatment groups [7]. To avoid this bias, we used a landmark approach by excluding all ICU admissions that were lost to follow-up within the first 24 hours after initiation, and started follow-up after the first 24 hours [7]. We conducted five sensitivity analyses for this exploratory analysis: subsetting the included admissions to the area of common support, no truncation of the weights, estimation without the landmark approach, estimation of the effect not mediated by competing events (by considering the competing events as censoring events and obtaining the weighted cumulative incidence curves using the Kaplan-Meier method) and multiple imputation by chained equations (using predictive mean matching to obtain five imputed datasets).

References

1. Lund JL, Richardson DB, Sturmer T. The active comparator, new user study design in pharmacoepidemiology: historical foundations and contemporary application. *Curr Epidemiol Rep*. Dec 2015;2(4):221-228. doi:10.1007/s40471-015-0053-5
2. van de Klundert N, Holman R, Dongelmans DA, de Keizer NF. Data Resource Profile: the Dutch National Intensive Care Evaluation (NICE) Registry of Admissions to Adult Intensive Care Units. *Int J Epidemiol*. Dec 2015;44(6):1850-1850h. doi:10.1093/ije/dyv291
3. Koeze J, Keus F, Dieperink W, van der Horst IC, Zijlstra JG, van Meurs M. Incidence, timing and outcome of AKI in critically ill patients varies with the definition used and the addition of urine output criteria. *BMC Nephrol*. Feb 20 2017;18(1):70. doi:10.1186/s12882-017-0487-8
4. Carrero JJ, Fu EL, Vestergaard SV, et al. Defining measures of kidney function in observational studies using routine health care data: methodological and reporting considerations. *Kidney Int*. Jan 2023;103(1):53-69. doi:10.1016/j.kint.2022.09.020
5. Wilson FP, Martin M, Yamamoto Y, et al. Electronic health record alerts for acute kidney injury: multicenter, randomized clinical trial. *BMJ*. Jan 18 2021;372:m4786. doi:10.1136/bmj.m4786
6. Patrick AR, Schneeweiss S, Brookhart MA, et al. The implications of propensity score variable selection strategies in pharmacoepidemiology: an empirical illustration. *Pharmacoepidemiol Drug Saf*. Jun 2011;20(6):551-9. doi:10.1002/pds.2098
7. Fu EL, van Diepen M, Xu Y, et al. Pharmacoepidemiology for nephrologists (part 2): potential biases and how to overcome them. *Clin Kidney J*. May 2021;14(5):1317-1326. doi:10.1093/ckj/sfaa242

Back to table of contents

Supplement S3. Target trial emulation protocol

Protocol component	Hypothetical trial	Limitations of our observational data and assumptions	Emulation
Eligibility criteria	Adult, non-dialysis dependent AKI-free ICU admissions with a suspected bacterial infection that is percieved to be treatable with vancomycin or one of the following alternative antibiotics: clindamycin, linezolid, teicoplanin, meropenem, cefazolin or daptomycin. Additionally, ICU admissions are only eligible after 24 hours and before seven days post ICU admission. Lastly, a baseline serum creatinine (SCr) measurement in the first 24 hours of the ICU admission is required.	In The Netherlands, when a drug is prescribed, it is not mandatory to register the indication. Therefore, when reusing electronic health record data in our context, no structured data on the suspected infection type linked to the administered antibiotics is present. To address this limitation, we **assume** that if one of the included antibiotics is initiated (as seen in the medication administration data), a bacterial infection was suspected and perceived to be treatable with this antibiotic. Very few admissions received linezolid, teicoplanin or daptomycin.	Same as in the hypothetical trial, except that we we assume that initiation of one of the study antibiotics reflects a suspected bacterial infection which was perceived to be treatable with the initiated antibiotic.
Treatment strategies	Initiate vancomycin or one of the alternative antibiotics: clindamycin, linezolid, teicoplanin, meropenem, cefazolin or daptomycin. Do not initiate the other treatment strategy after initiation. Follow standard therapy procedures for the respective initiated antibiotic (e.g. until 48 hours post negative blood culture).		Same as in the hypothetical trial.
Assignment procedure	Randomized, non-blinded assignment into one of the treatment strategies when eligibility criteria are met.	Treatment assignment is not random, but based on admission characteristics. No data available on pre-ICU prevalent users. Unclear timing of some admission characteristics in the first 24 hours of the ICU admission (e.g. APACHE IV mortality probability). Number of admissions in the ICU decreases over time, so lower counts of vancomycin and alternative initiations as length of stay increases. A**ssume** conditional exchangeability. Don't include admissions if treatment is initiated within the first 24 hours of the ICU admission (washout period) or later than 7 days after ICU admission (both part of the hypothetical trial's eligibility criteria). **Assume** that the remaining eligible ICU admissions are new users as they initiated the treatment strategy after the first 24 hours of the ICU admission. **Assume** that the time between the suspected infection and initiation of the treatment is equal for vancomycin and the alternatives.	ICU admissions are assigned to the treatment strategies according to the observational data. ICU admissions that initiated vancomycin and an alternative antibiotic simultaneously according to the observational data are excluded.<br><br>Randomization is emulated through inverse probability of treatment weighting (IPTW).
Follow-up period	Follow-up starts at the time of assignment to a treatment strategy (and all eligibility criteria are met) and ends at AKI diagnosis, loss to follow-up (e.g., when transferred to a non-participating hospital), initiation of the other treatment strategy, initiation of kidney replacement therapy (KRT, without preceding AKI), death or 14 days after treatment assignment, whichever occurs first.	No post-ICU follow-up data.	Follow-up starts at initiation of treatment, and ends at AKI diagnosis, discharge from the ICU (e.g., when transferred to a non-ICU ward within the same hospital), initiation of the other treatment strategy, initiation of KRT (without preceding AKI), death or 14 days after treatment initiation, whichever occurs first.

Back to table of contents

Outcome	AKI according to the KDIGO SCr or urine output (UO) criteria.	Relative SCr increase should be calculated using a historical SCr baseline, but not available in our data. Longitudinal UO data after the first 24 hours of the ICU admission is often missing and heterogeneous across ICUs.	AKI according to the KDIGO SCr criteria. Use the first SCr in the first 24 hours of the ICU admission as the SCr baseline.
Causal contrasts of interest	Per protocol, average treatment effect. The estimands of interest are the absolute risks and risk differences at days 2 and 14 of follow-up.	Positivity must be assessed to ascertain a nonzero probability of assignment to each treatment strategy. **Assume** positivity holds after ascertaining sufficient overlap in the treatment probability distributions.	Same as in the hypothetical trial.
Analysis plan	Non-naïve per protocol analysis. ICU admissions are censored at loss to follow-up, initiation of the other treatment strategy and 14 days after treatment initiation.<br><br>Weighted cumulative incidence curves are estimated for each treatment strategy using the Aalen-Johansen method while accounting for two competing risks: initiation of KRT (without a preceding AKI) and death. We apply inverse probability of censoring weighting (IPCW) and IPTW to adjust for selection and confounding bias, respectively. The time-varying weights for IPCW are calculated using pooled logistic regression, whilst the weights for IPTW are calculated using logistic regression.<br><br>Cluster bootstrapping with 1,000 replications is used to obtain 95% confidence intervals.<br><br>**Assume** stable unit of treatment value (SUTVA), positivity and conditional exchangeability for IPCW and IPTW.	Treatment effect might depend on treatment assignment in other admissions (e.g. due to antibiotic resistance). Missing data. **Assume** SUTVA.	Per protocol analysis as in the analysis plan for the hypothetical trial. ICU admissions are additionally censored at ICU discharge.<br><br>Missing data in static variables are imputed using the mean or median (for continuous and categorical data, respectively). Missing data in longitudinal variables are imputed using forward filling. If admission values are missing for the longitidinal variables, these are first imputed by the mean or median, followed by forward filling.

Back to table of contents

Supplement S4. Potential confounders

Potential confounder	Factor type	Static / longitudinal	Data type	Availability	Data source	Variable name or variable construction description
Acute heart failure	Acute	Static	Binary	Indirect	NICE MDS [Diagnoses on ICU admission] & [Diagnosis following first 24 hours of ICU admission] & [Admission and Patient Details]	CPR OR thrombolytic therapy following acute myocardial infarction OR number of CABG grafts > 0 OR internal mammary arterial graft OR APACHE IV admission diagnoses for CABG or AHF. Only scored if admission was not a planned surgery admission
Burns	Acute	Static	Binary	Direct	NICE MDS [Diagnosis on ICU admission]	Burns
Graft and transplant surgery	Acute	Static	Binary	Indirect	NICE MDS [Diagnosis on ICU admission] & [Diagnosis following first 24 hours of ICU admission]	Number of CABG grafts > 0 OR internal mammary arterial graft OR APACHE IV admission diagnoses for grafting or organ transplantation
Hypoalbuminemia	Acute	Longitudinal	Binary	Indirect	RESCUE data [Lab]	Serum albumin < 3.0 g/dL (during each 24 hour period)
Hypotension	Acute	Longitudinal	Binary	Indirect	RESCUE data [ABP measurements]	MAP < 60 mmHG in ≥ 2 successive measurements (during each 24 hour period)[a]
Hypovolemia	Acute	Static	Binary	Indirect	NICE MDS [Diagnosis on ICU admission] & [Diagnosis following first 24 hours of ICU admission]	Gastrointestinal bleeding OR APACHE IV admission diagnoses for hemorrhage or hypovolemia
Major surgery	Acute	Static	Binary	Indirect	NICE MDS [Diagnosis following first 24 hours of ICU admission]	APACHE IV admission diagnoses for major surgery (expert opinion classification)
Mechanical ventilation	Acute	Static	Binary	Indirect	NICE MDS [Diagnosis on ICU admission] & [Diagnosis following first 24 hours of ICU admission]	Mechanical ventilation on ICU admission OR mechanical ventilation within 24 hours
Sepsis	Acute	Static	Binary	Indirect	NICE MDS [Diagnosis following first 24 hours of ICU admission] & [Physiology and Laboratory details] & [Glasgow Coma Scale components]	APACHE IV admission diagnoses for sepsis OR day 1 SOFA score >= 2 combined with a confirmed infection within 24 hours of admission[b]
Sepsis	Acute	Longitudinal	Binary	Indirect	NICE MDS [Diagnosis following first 24 hours of ICU admission] & NICE SOFA	SOFA score >= 2 combined with a confirmed infection within 24 hours of admission (during each 24 hour period)
Trauma	Acute	Static	Binary	Indirect	NICE MDS [Diagnosis following first 24 hours of ICU admission]	APACHE IV admission diagnoses for trauma
Age	Demographic	Static	Integer	Direct	NICE MDS [Admission and Patient Details]	Age
Alcohol abuse	Chronic	Static	Binary	Indirect	RESCUE data [Drug administrations]	Thiamine administration during ICU admission
Cardiovascular disease	Chronic	Static	Binary	Indirect	NICE MDS [Diagnosis on ICU admission] & [Chronic Diagnoses]	Chronic cardiovascular insufficiency (NYHA IV) OR myocardial infarction before ICU admission OR number of grafts > 0 OR internal mammary arterial graft OR pre-operative ejection fraction < 40%[c]
Chronic kidney disease	Chronic	Static	Binary	Indirect	NICE MDS [Chronic Diagnoses]	Chronic renal insufficiency OR chronic dialysis
Chronic pulmonary disease	Chronic	Static	Binary	Indirect	NICE MDS [Chronic Diagnoses]	Chronic obstructive pulmonary disease OR chronic respiratory insufficiency
Diabetes mellitus	Chronic	Static	Binary	Direct	NICE MDS [Diagnosis on ICU admission]	Diabetes
Hypertension	Chronic	Static	NA	Unavailable	NA	NA
Liver disease	Chronic	Static	Binary	Direct	NICE MDS [Chronic Diagnoses]	Cirrhosis
Malignancy	Chronic	Static	Binary	Indirect	NICE MDS [Chronic Diagnoses] & [Diagnosis following first 24 hours of ICU admission]	Metastasized neoplasm OR haematological malignancy OR APACHE IV admission diagnoses for malignancy
Obesity	Chronic	Static	Binary	Indirect	NICE MDS [Admission and Patient Details]	BMI ≥ 30 (calculated using weight and height variables)
Sex	Demographic	Static	Binary	Direct	NICE MDS [Admission and Patient Details]	Gender
Smoking	Chronic	Static	NA	Unavailable	NA	NA
SCr baseline	Other	Static	Continuous	Indirect	RESCUE data [Lab]	First SCr value within first 24 hours of ICU admission
eGFR	Other	Static	Continuous	Indirect	RESCUE data [Lab]	MDRD formula using SCr baseline, age and sex
SCr trend	Other	Static	Continuous	Indirect	RESCUE data [Lab]	Change in SCr in mg/dL per 3 days before vancomycin / alternative antibiotic initiation
APACHE IV mortality probability	Other	Static	Continuous	Direct	NICE MDS	Calculated by NICE Registry Research & Support
ICU	Other	Static	Character	Direct	RESCUE data	ICU identifier
Nephrotoxins[d]	Acute	Longitudinal	Binary	Direct	RESCUE data [Drug administrations]	Exposed / not exposed (during each 24 hour period)

Potential confounder	Factor type	Static / longitudinal	Data type	Availability	Data source	Variable name or variable construction description
SCr	Acute	Longitudinal	Continuous	Direct	RESCUE data [Lab]	Max (during each 24 hour period)
Serum albumin	Acute	Longitudinal	Continuous	Direct	RESCUE data [Lab]	Min (during each 24 hour period)
Leukocytes	Acute	Longitudinal	Continuous	Direct	RESCUE data [Lab]	Max (during each 24 hour period)
MAP	Acute	Longitudinal	Continuous	Direct	RESCUE data [ABP measurements]	Min (during each 24 hour period)
SOFA score	Acute	Longitudinal	Integer	Direct	NICE SOFA	Max (during each 24 hour period)
UO rate	Acute	Longitudinal	Continuous	Direct	NICE SOFA	Min (during each 24 hour period)
Temperature	Acute	Longitudinal	Continuous	Direct	RESCUE data [Temperature]	Max (during each 24 hour period)

AKI: acute kidney injury, NICE: National Intensive Care Evaluation quality registry, MDS: minimal dataset, ICU: intensive care unit, CPR: cardiopulmonary resuscitation, CABG: coronary artery bypass grafting, APACHE IV: Acute Physiology and Chronic Health Evaluation IV, AHF: acute heart failure, RESCUE: Towards a leaRning mEdication Safety system in a national network of Intensive Care Units – timely detection of adverse drug Events, ABP: arterial blood pressure, MAP: mean arterial pressure, SOFA: sequential organ failure assessment, NYHA IV: New York Heart Association IV, BMI: Body mass index, SCr: serum creatinine, eGFR: estimated glomerular filtration rate, UO: urine output.
[a]Lehman et al. (2010) [1]
[b]Singer et al. (2016) [2]
[c]Ponikowski et al. (2016) [3]
[d]See Supplement S5

References

1. Lehman LW, Saeed M, Moody G, Mark R. Hypotension as a risk factor for acute kidney injury in ICU patients. *2010 Computing in Cardiology*. 2010:1095-1098.
2. Singer M, Deutschman CS, Seymour CW, et al. The Third International Consensus Definitions for Sepsis and Septic Shock (Sepsis-3). *JAMA*. Feb 23 2016;315(8):801-10. doi:10.1001/jama.2016.0287
3. Ponikowski P, Voors AA, Anker SD, et al. 2016 ESC Guidelines for the diagnosis and treatment of acute and chronic heart failure: The Task Force for the diagnosis and treatment of acute and chronic heart failure of the European Society of Cardiology (ESC). Developed with the special contribution of the Heart Failure Association (HFA) of the ESC. *European Journal of Heart Failure*. Aug 2016;18(8):891-975. doi:10.1002/ejhf.592

Back to table of contents

Supplement S5. Potential nephrotoxic drugs

ATC	Drug name
J05AF06	Abacavir
J05AB01	Aciclovir
J05AF08	Adefovir dipivoxil
L01XX44	Aflibercept
L03AC01	Aldesleukin
L01XE36	Alectinib
L04AA34	Alemtuzumab
L01XX22	Alitretinoin
L01XX65	Alpelisib
L01XX03	Altretamine
J01GB06	Amikacin
J02AA01	Amphotericin B
L01XX01	Amsacrine
L01XX35	Anagrelide
J02AX06	Anidulafungin
L01XX27	Arsenic trioxide
L01XX02	Asparaginase
L01XC31	Avelumab
L01XE17	Axitinib
L01BC07	Azacitidine
M03BX01	Baclofen
L04AC02	Basiliximab
L03AX03	BCG vaccine
L04AA28	Belatacept
L01XX49	Belinostat
L01XX68	Belotecan
L01AA09	Bendamustine
L01XC07	Bevacizumab
L01XX25	Bexarotene
L01XE41	Binimetinib
L01XX32	Bortezomib
L01XE14	Bosutinib
L01XE43	Brigatinib
L01AB01	Busulfan
L01CD04	Cabazitaxel
L01XE26	Cabozantinib
L04AC08	Canakinumab
C09CA06	Candesartan
L01XA02	Carboplatin
L01XX45	Carfilzomib
C07AG02	Carvedilol
J02AX04	Caspofungin
M01AH01	Celecoxib
L01XX33	Celecoxib
L01XC33	Cemiplimab
L01XE28	Ceritinib
L04AD01	Ciclosporin
J05AB12	Cidofovir
A02BA01	Cimetidine
L01XA01	Cisplatin
C08CA16	Clevidipine
L01BB06	Clofarabine
N05AH02	Clozapine
M04AC01	Colchicine
J01XB01	Colistin
L01XX61	Copanlisib
L01XE16	Crizotinib
L01AA01	Cyclophosphamide
L01BC01	Cytarabine
L01XY01	Cytarabine and daunorubicin
A10BK01	Dapagliflozin
V03AC03	Deferasirox
L01XX29	Denileukin diftitox
M01AB05	Diclofenac
V03AB24	Digitalis antitoxin
L04AX07	Dimethyl fumarate
L01XC16	Dinutuximab beta
L01XC28	Durvalumab
B02BX05	Eltrombopag
J05AR09	Emtricitabine, tenofovir disoproxil, elvitegravir and cobicistat
C09AA02	Enalapril
L01XX59	Enasidenib
L01XE46	Encorafenib
L01XX64	Entinostat
L01XX58	Epacadostat
C03DA04	Eplerenone
L01XX41	Eribulin
L01XE03	Erlotinib
A10BK04	Ertugliflozin
L01XX11	Estramustine
L01XX56	Etirinotecan pegol
J05AG04	Etravirine
L01XE10	Everolimus
L04AA18	Everolimus
L03AA02	Filgrastim
J05AD01	Foscarnet
C03CA01	Furosemide
J05AB06	Ganciclovir
L01XE02	Gefitinib
L01BC05	Gemcitabine
J01GB03	Gentamicin

Back to table of contents

L01XX63	Glasdegib
J05AP57	Glecaprevir and pibrentasvir
C03AA03	Hydrochlorothiazide
L01XX05	Hydroxycarbamide
M01AE01	Ibuprofen
L01XX47	Idelalisib
L01AA06	Ifosfamide
J05AE02	Indinavir
M01AB01	Indometacin
N07XX15	Inotersen
L03AB03	Interferon gamma
L01XX19	Irinotecan
J02AC05	Isavuconazole
D10BA01	Isotretinoin
L01XX62	Ivosidenib
L01XX50	Ixazomib
M01AE03	Ketoprofen
M01AB15	Ketorolac
L04AX04	Lenalidomide
L01XE29	Lenvatinib
M04AB05	Lesinurad
L02AE02	Leuprorelin
C09AA03	Lisinopril
N05AN01	Lithium
L01XX07	Lonidamine
C09CA01	Losartan
N05AE05	Lurasidone
V10XX04	Lutetium (177Lu) oxodotreotide
L01XX10	Masoprocol
A07EC02	Mesalazine
V03AF01	Mesna
L04AX03	Methotrexate
P01CX04	Miltefosine
L01XX16	Mitoguazone
L01DC03	Mitomycin
L01XX23	Mitotane
L04AA06	Mycophenolic acid
M01AE02	Naproxen
L01BB07	Nelarabine
L01XE45	Neratinib
L01XX54	Niraparib
L01XC17	Nivolumab
L01XX36	Oblimersen
L01XX46	Olaparib
C09CA08	Olmesartan medoxomil
L01XX40	Omacetaxine mepesuccinate
M05BA03	Pamidronic acid
L01XX42	Panobinostat
L01XX24	Pegaspargase
P01CX01	Pentamidine isethionate
L01XX08	Pentostatin
L01XX57	Plitidepsin
C09AA06	Quinapril
J05AE03	Ritonavir
B01AF01	Rivaroxaban
M01AH02	Rofecoxib
L01XX39	Romidepsin
L01XX55	Rucaparib
L01XX66	Selinexor
L04AA10	Sirolimus
L01XX37	Sitimagene ceradenovec
M01CB01	Sodium aurothiomalate
A16AX03	Sodium phenylbutyrate
H01AC01	Somatropin
L01XX48	Sonidegib
J01GA01	Streptomycin
J01EE01	Sulfamethoxazole and trimethoprim
A07EC01	Sulfasalazine
L04AD02	Tacrolimus
L01XX67	Tagraxofusp
L01XX60	Talazoparib
L01XX51	Talimogene laherparepvec
L01XX18	Tiazofurine
B01AC24	Ticagrelor
J01AA12	Tigecycline
J01GB01	Tobramycin
L01XX17	Topotecan
L01XX14	Tretinoin
J05AB11	Valaciclovir
J05AB14	Valganciclovir
C09CA03	Valsartan
L01XE15	Vemurafenib
L01XX52	Venetoclax
L01XX43	Vismodegib
J02AC03	Voriconazole
L01XX38	Vorinostat
L01XX53	Vosaroxin

Back to table of contents

Supplement S6. Extended baseline characteristics and crude outcomes – primary analysis

Characteristic	Alternative (n = 922)	Vancomycin (n = 887)
Arm options		
J01XA01 - Vancomycin, No. (%)	0 (0.0)	887 (100.0)
J01FF01 - Clindamycin, No. (%)	206 (22.3)	0 (0.0)
J01XX08 - Linezolid, No. (%)	20 (2.2)	0 (0.0)
J01XA02 - Teicoplanin, No. (%)	2 (0.2)	0 (0.0)
J01DH02 - Meropenem, No. (%)	337 (36.6)	0 (0.0)
J01DB04 - Cefazolin, No. (%)	355 (38.5)	0 (0.0)
J01XX09 - Daptomycin, No. (%)	2 (0.2)	0 (0.0)
Age (Years), median (Q1 - Q3)	63.0 (52.0 - 72.0)	65.0 (54.0 - 73.0)
Male sex, No. (%)	555 (60.2)	549 (61.9)
Planned admission, No. (%)	92 (10.0)	135 (15.2)
Admission type		
Medical, No. (%)	677 (73.5)	551 (62.1)
Emergency surgical, No. (%)	151 (16.4)	191 (21.5)
Elective surgical, No. (%)	93 (10.1)	145 (16.3)
APACHE IV score, median (Q1 - Q3)	67.0 (48.0 - 83.0)	69.0 (56.0 - 85.5)
APACHE IV mortality probability, median (Q1 - Q3)	0.2 (0.1 - 0.4)	0.3 (0.1 - 0.4)
SCr baseline (mg/dL), median (Q1 - Q3)	0.9 (0.7 - 1.2)	0.9 (0.7 - 1.2)
eGFR baseline (mL/min/1.73 m²), median (Q1 - Q3)	82.1 (55.5 - 108.1)	80.3 (56.8 - 110.4)
Highest SCr during admission (mg/dL), median (Q1 - Q3)	0.9 (0.7 - 1.2)	1.0 (0.7 - 1.2)
SCr trend during admission (mg/dL per 3 days), median (Q1 - Q3)	-0.2 (-0.3 - 0.0)	-0.1 (-0.3 - 0.0)
Lowest urine output during admission (mL/kg/h), median (Q1 - Q3)	0.9 (0.7 - 1.3)	0.9 (0.7 - 1.3)
Lowest serum albumin during admission (g/dL), median (Q1 - Q3)	2.5 (2.0 - 2.7)	2.4 (1.8 - 2.5)
Lowest MAP during admission (mmHg), median (Q1 - Q3)	57.0 (50.0 - 63.0)	56.0 (49.0 - 61.0)
Highest SOFA score during admission, median (Q1 - Q3)	7.0 (5.0 - 10.0)	8.0 (6.0 - 10.0)
Highest temperature during admission (°C), median (Q1 - Q3)	38.3 (37.7 - 39.0)	38.6 (37.9 - 39.4)

Back to table of contents

Highest leukocyte count during admission (10**9 cells/L), median (Q1 - Q3)	16.5 (11.9 - 21.8)	17.1 (11.9 - 23.0)
Acute AKI risk factors		
Acute heart failure, No. (%)	102 (11.1)	117 (13.2)
Burns, No. (%)	1 (0.1)	0 (0.0)
Graft or transplant surgery, No. (%)	46 (5.0)	71 (8.0)
Hypoalbuminemia, No. (%)	777 (84.3)	782 (88.2)
Hypotension, No. (%)	310 (33.6)	341 (38.4)
Hypovolemia, No. (%)	23 (2.5)	25 (2.8)
Major surgery, No. (%)	209 (22.7)	269 (30.3)
Mechanical ventilation, No. (%)	586 (63.6)	668 (75.3)
Sepsis - admission diagnosis, No. (%)	141 (15.3)	151 (17.0)
Sepsis - longitudinal, No. (%)	326 (35.4)	374 (42.2)
Trauma, No. (%)	96 (10.4)	25 (2.8)
Chronic AKI risk factors		
Alcohol abuse, No. (%)	193 (20.9)	214 (24.1)
Cardiovascular disease, No. (%)	272 (29.5)	221 (24.9)
Chronic kidney disease, No. (%)	12 (1.3)	24 (2.7)
Chronic pulmonary disease, No. (%)	159 (17.2)	118 (13.3)
Diabetes mellitus, No. (%)	138 (15.0)	136 (15.3)
Liver disease, No. (%)	12 (1.3)	13 (1.5)
Malignancy, No. (%)	115 (12.5)	160 (18.0)
Obesity, No. (%)	128 (13.9)	140 (15.8)
APACHE IV admission diagnosis category		
Cardiovascular, No. (%)	275 (29.8)	288 (32.5)
Gastrointestinal, No. (%)	154 (16.7)	248 (28.0)
Genitourinary, No. (%)	8 (0.9)	12 (1.4)
Hematology, No. (%)	6 (0.7)	14 (1.6)
Metabolic/Endocrine, No. (%)	14 (1.5)	8 (0.9)
Musculoskeletal/Skin, No. (%)	9 (1.0)	7 (0.8)
Neurologic, No. (%)	97 (10.5)	64 (7.2)

Back to table of contents

Respiratory, No. (%)	260 (28.2)	220 (24.8)
Transplant, No. (%)	10 (1.1)	3 (0.3)
Trauma, No. (%)	89 (9.7)	22 (2.5)
Nephrotoxin exposure		
A07EC01 - sulfasalazine, No. (%)	0 (0.0)	1 (0.1)
A07EC02 - mesalazine, No. (%)	0 (0.0)	3 (0.3)
B01AC24 - ticagrelor, No. (%)	17 (1.8)	14 (1.6)
C03AA03 - hydrochlorothiazide, No. (%)	27 (2.9)	31 (3.5)
C03CA01 - furosemide, No. (%)	336 (36.4)	482 (54.3)
C07AG02 - carvedilol, No. (%)	1 (0.1)	3 (0.3)
C09AA02 - enalapril, No. (%)	7 (0.8)	10 (1.1)
C09AA03 - lisinopril, No. (%)	8 (0.9)	14 (1.6)
C09CA01 - losartan, No. (%)	5 (0.5)	4 (0.5)
C09CA03 - valsartan, No. (%)	7 (0.8)	2 (0.2)
C09CA06 - candesartan, No. (%)	1 (0.1)	0 (0.0)
J01EE01 - sulfamethoxazole and trimethoprim, No. (%)	38 (4.1)	57 (6.4)
J01GB01 - tobramycin, No. (%)	14 (1.5)	14 (1.6)
J01GB03 - gentamicin, No. (%)	140 (15.2)	149 (16.8)
J01GB06 - amikacin, No. (%)	0 (0.0)	1 (0.1)
J01XB01 - colistin, No. (%)	7 (0.8)	2 (0.2)
J02AA01 - amphotericin B, No. (%)	9 (1.0)	27 (3.0)
J02AC03 - voriconazole, No. (%)	14 (1.5)	25 (2.8)
J02AX04 - caspofungin, No. (%)	4 (0.4)	11 (1.2)
J02AX06 - anidulafungin, No. (%)	2 (0.2)	11 (1.2)
J05AB01 - aciclovir, No. (%)	10 (1.1)	27 (3.0)
J05AB06 - ganciclovir, No. (%)	9 (1.0)	11 (1.2)
J05AB11 - valaciclovir, No. (%)	13 (1.4)	15 (1.7)
J05AB14 - valganciclovir, No. (%)	1 (0.1)	0 (0.0)
J05AD01 - foscarnet, No. (%)	1 (0.1)	1 (0.1)
J05AE03 - ritonavir, No. (%)	0 (0.0)	1 (0.1)

L01AA01 - cyclophosphamide, No. (%)	0 (0.0)	1 (0.1)
L01BC01 - cytarabine, No. (%)	1 (0.1)	0 (0.0)
L01XA01 - cisplatin, No. (%)	0 (0.0)	1 (0.1)
L01XX05 - hydroxycarbamide, No. (%)	0 (0.0)	2 (0.2)
L03AA02 - filgrastim, No. (%)	2 (0.2)	13 (1.5)
L04AA06 - mycophenolic acid, No. (%)	14 (1.5)	13 (1.5)
L04AA18 - everolimus, No. (%)	0 (0.0)	1 (0.1)
L04AC02 - basiliximab, No. (%)	6 (0.7)	1 (0.1)
L04AD01 - ciclosporin, No. (%)	4 (0.4)	6 (0.7)
L04AD02 - tacrolimus, No. (%)	11 (1.2)	8 (0.9)
M01AB05 - diclofenac, No. (%)	25 (2.7)	11 (1.2)
M01AE01 - ibuprofen, No. (%)	1 (0.1)	0 (0.0)
M01AE02 - naproxen, No. (%)	1 (0.1)	0 (0.0)
M01AH01 - celecoxib, No. (%)	0 (0.0)	1 (0.1)
M03BX01 - baclofen, No. (%)	12 (1.3)	9 (1.0)
M04AC01 - colchicine, No. (%)	0 (0.0)	3 (0.3)
M05BA03 - pamidronic acid, No. (%)	1 (0.1)	1 (0.1)
N05AH02 - clozapine, No. (%)	2 (0.2)	4 (0.5)
N05AN01 - lithium, No. (%)	4 (0.4)	2 (0.2)
Time in ICU before initiation (Days), median (Q1 - Q3)	2.1 (1.4 - 3.5)	2.6 (1.7 - 4.3)
AKI, No. (%)	125 (13.6)	167 (18.8)
AKI stage		
Stage 1, No. (%)	85 (68.0)	106 (63.5)
Stage 2, No. (%)	14 (11.2)	27 (16.2)
Stage 3, No. (%)	26 (20.8)	34 (20.4)
KRT, No. (%)	22 (17.6)	23 (13.8)
Censoring events and competing risks		
Discharged alive from the ICU, No. (%)	571 (61.9)	477 (53.8)
Initiation of other treatment option, No. (%)	114 (12.4)	81 (9.1)
KRT without preceding AKI, No. (%)	2 (0.2)	9 (1.0)

Back to table of contents

Death in the ICU, No. (%)	43 (4.7)	49 (5.5)
Total ICU length of stay (Days), median (Q1 - Q3)	6.8 (3.8 - 12.9)	8.7 (4.9 - 16.8)
Total ICU mortality, No. (%)	122 (13.2)	147 (16.6)
Total hospital mortality, No. (%)	171 (18.5)	210 (23.7)

Back to table of contents

Supplement S7. Extended baseline characteristics and crude outcomes with missing data – primary analysis

Characteristic	Alternative (n = 922)	Vancomycin (n = 887)
Arm options		
J01XA01 - Vancomycin, No. (%)	0 (0.0)	887 (100.0)
J01FF01 - Clindamycin, No. (%)	206 (22.3)	0 (0.0)
J01XX08 - Linezolid, No. (%)	20 (2.2)	0 (0.0)
J01XA02 - Teicoplanin, No. (%)	2 (0.2)	0 (0.0)
J01DH02 - Meropenem, No. (%)	337 (36.6)	0 (0.0)
J01DB04 - Cefazolin, No. (%)	355 (38.5)	0 (0.0)
J01XX09 - Daptomycin, No. (%)	2 (0.2)	0 (0.0)
Age (Years), median (Q1 - Q3)	63.0 (52.0 - 72.0)	65.0 (54.0 - 73.0)
Missing, %	0	0
Male sex, No. (%)	555 (60.2)	549 (61.9)
Missing, %	0	0
Planned admission, No. (%)	92 (10.0)	135 (15.2)
Missing, %	0	0
Admission type		
Medical, No. (%)	677 (73.5)	551 (62.1)
Emergency surgical, No. (%)	151 (16.4)	191 (21.5)
Elective surgical, No. (%)	93 (10.1)	145 (16.3)
Missing, %	0	0
APACHE IV score, median (Q1 - Q3)	67.0 (48.0 - 83.0)	69.0 (56.0 - 85.5)
Missing, %	0	0
APACHE IV mortality probability, median (Q1 - Q3)	0.2 (0.1 - 0.4)	0.2 (0.1 - 0.5)
Missing, %	1	1
SCr baseline (mg/dL), median (Q1 - Q3)	0.9 (0.7 - 1.2)	0.9 (0.7 - 1.2)
Missing, %	0	0
eGFR baseline (mL/min/1.73 m²), median (Q1 - Q3)	82.1 (55.5 - 108.1)	80.3 (56.8 - 110.4)
Missing, %	0	0

Back to table of contents

Highest SCr during admission (mg/dL), median (Q1 - Q3)	0.9 (0.7 - 1.2)	1.0 (0.7 - 1.2)
Missing, %	0	0
SCr trend during admission (mg/dL per 3 days), median (Q1 - Q3)	-0.1 (-0.4 - 0.0)	-0.1 (-0.3 - 0.1)
Missing, %	6	3
Lowest urine output during admission (mL/kg/h), median (Q1 - Q3)	0.9 (0.7 - 1.3)	0.9 (0.7 - 1.4)
Missing, %	3	2
Lowest serum albumin during admission (g/dL), median (Q1 - Q3)	2.4 (1.9 - 2.9)	2.2 (1.7 - 2.7)
Missing, %	24	15
Lowest MAP during admission (mmHg), median (Q1 - Q3)	57.0 (49.5 - 63.0)	56.0 (49.0 - 61.0)
Missing, %	1	0
Highest SOFA score during admission, median (Q1 - Q3)	7.0 (5.0 - 10.0)	8.0 (6.0 - 10.0)
Missing, %	0	0
Highest temperature during admission (°C), median (Q1 - Q3)	38.3 (37.7 - 39.1)	38.6 (37.9 - 39.4)
Missing, %	2	0
Highest leukocyte count during admission (10**9 cells/L), median (Q1 - Q3)	16.0 (11.7 - 22.0)	16.9 (11.8 - 23.1)
Missing, %	2	1
Acute AKI risk factors		
Acute heart failure, No. (%)	102 (11.1)	117 (13.2)
Missing, %	0	0
Burns, No. (%)	1 (0.1)	0 (0.0)
Missing, %	0	0
Graft or transplant surgery, No. (%)	46 (5.0)	71 (8.0)
Missing, %	0	0
Hypoalbuminemia, No. (%)	555 (60.2)	636 (71.7)
Missing, %	24	15
Hypotension, No. (%)	310 (33.6)	341 (38.4)
Missing, %	1	0
Hypovolemia, No. (%)	23 (2.5)	25 (2.8)
Missing, %	0	0
Major surgery, No. (%)	209 (22.7)	269 (30.3)

Back to table of contents

Missing, %	0	0
Mechanical ventilation, No. (%)	586 (63.6)	668 (75.3)
Missing, %	0	0
Sepsis - admission diagnosis, No. (%)	141 (15.3)	151 (17.0)
Missing, %	0	0
Sepsis - longitudinal, No. (%)	326 (35.4)	374 (42.2)
Missing, %	0	0
Trauma, No. (%)	96 (10.4)	25 (2.8)
Missing, %	0	0
Chronic AKI risk factors		
Alcohol abuse, No. (%)	193 (20.9)	214 (24.1)
Missing, %	0	0
Cardiovascular disease, No. (%)	272 (29.5)	221 (24.9)
Missing, %	0	0
Chronic kidney disease, No. (%)	12 (1.3)	24 (2.7)
Missing, %	0	0
Chronic pulmonary disease, No. (%)	159 (17.2)	118 (13.3)
Missing, %	0	0
Diabetes mellitus, No. (%)	138 (15.0)	136 (15.3)
Missing, %	0	0
Liver disease, No. (%)	12 (1.3)	13 (1.5)
Missing, %	0	0
Malignancy, No. (%)	115 (12.5)	160 (18.0)
Missing, %	0	0
Obesity, No. (%)	128 (14.3)	140 (16.1)
Missing, %	3	2
APACHE IV admission diagnosis category		
Cardiovascular, No. (%)	275 (29.8)	288 (32.5)
Gastrointestinal, No. (%)	154 (16.7)	248 (28.0)
Genitourinary, No. (%)	8 (0.9)	12 (1.4)

Hematology, No. (%)	6 (0.7)	14 (1.6)
Metabolic/Endocrine, No. (%)	14 (1.5)	8 (0.9)
Musculoskeletal/Skin, No. (%)	9 (1.0)	7 (0.8)
Neurologic, No. (%)	97 (10.5)	64 (7.2)
Respiratory, No. (%)	260 (28.2)	220 (24.8)
Transplant, No. (%)	10 (1.1)	3 (0.3)
Trauma, No. (%)	89 (9.7)	22 (2.5)
Missing, %	0	0
Nephrotoxin exposure		
A07EC01 - sulfasalazine, No. (%)	0 (0.0)	1 (0.1)
A07EC02 - mesalazine, No. (%)	0 (0.0)	3 (0.3)
B01AC24 - ticagrelor, No. (%)	17 (1.8)	14 (1.6)
C03AA03 - hydrochlorothiazide, No. (%)	27 (2.9)	31 (3.5)
C03CA01 - furosemide, No. (%)	336 (36.4)	482 (54.3)
C07AG02 - carvedilol, No. (%)	1 (0.1)	3 (0.3)
C09AA02 - enalapril, No. (%)	7 (0.8)	10 (1.1)
C09AA03 - lisinopril, No. (%)	8 (0.9)	14 (1.6)
C09CA01 - losartan, No. (%)	5 (0.5)	4 (0.5)
C09CA03 - valsartan, No. (%)	7 (0.8)	2 (0.2)
C09CA06 - candesartan, No. (%)	1 (0.1)	0 (0.0)
J01EE01 - sulfamethoxazole and trimethoprim, No. (%)	38 (4.1)	57 (6.4)
J01GB01 - tobramycin, No. (%)	14 (1.5)	14 (1.6)
J01GB03 - gentamicin, No. (%)	140 (15.2)	149 (16.8)
J01GB06 - amikacin, No. (%)	0 (0.0)	1 (0.1)
J01XB01 - colistin, No. (%)	7 (0.8)	2 (0.2)
J02AA01 - amphotericin B, No. (%)	9 (1.0)	27 (3.0)
J02AC03 - voriconazole, No. (%)	14 (1.5)	25 (2.8)
J02AX04 - caspofungin, No. (%)	4 (0.4)	11 (1.2)
J02AX06 - anidulafungin, No. (%)	2 (0.2)	11 (1.2)
J05AB01 - aciclovir, No. (%)	10 (1.1)	27 (3.0)

Back to table of contents

J05AB06 - ganciclovir, No. (%)	9 (1.0)	11 (1.2)
J05AB11 - valaciclovir, No. (%)	13 (1.4)	15 (1.7)
J05AB14 - valganciclovir, No. (%)	1 (0.1)	0 (0.0)
J05AD01 - foscarnet, No. (%)	1 (0.1)	1 (0.1)
J05AE03 - ritonavir, No. (%)	0 (0.0)	1 (0.1)
L01AA01 - cyclophosphamide, No. (%)	0 (0.0)	1 (0.1)
L01BC01 - cytarabine, No. (%)	1 (0.1)	0 (0.0)
L01XA01 - cisplatin, No. (%)	0 (0.0)	1 (0.1)
L01XX05 - hydroxycarbamide, No. (%)	0 (0.0)	2 (0.2)
L03AA02 - filgrastim, No. (%)	2 (0.2)	13 (1.5)
L04AA06 - mycophenolic acid, No. (%)	14 (1.5)	13 (1.5)
L04AA18 - everolimus, No. (%)	0 (0.0)	1 (0.1)
L04AC02 - basiliximab, No. (%)	6 (0.7)	1 (0.1)
L04AD01 - ciclosporin, No. (%)	4 (0.4)	6 (0.7)
L04AD02 - tacrolimus, No. (%)	11 (1.2)	8 (0.9)
M01AB05 - diclofenac, No. (%)	25 (2.7)	11 (1.2)
M01AE01 - ibuprofen, No. (%)	1 (0.1)	0 (0.0)
M01AE02 - naproxen, No. (%)	1 (0.1)	0 (0.0)
M01AH01 - celecoxib, No. (%)	0 (0.0)	1 (0.1)
M03BX01 - baclofen, No. (%)	12 (1.3)	9 (1.0)
M04AC01 - colchicine, No. (%)	0 (0.0)	3 (0.3)
M05BA03 - pamidronic acid, No. (%)	1 (0.1)	1 (0.1)
N05AH02 - clozapine, No. (%)	2 (0.2)	4 (0.5)
N05AN01 - lithium, No. (%)	4 (0.4)	2 (0.2)
Time in ICU before initiation (Days), median (Q1 - Q3)	2.1 (1.4 - 3.5)	2.6 (1.7 - 4.3)
AKI, No. (%)	125 (13.6)	167 (18.8)
AKI stage		
Stage 1, No. (%)	85 (68.0)	106 (63.5)
Stage 2, No. (%)	14 (11.2)	27 (16.2)
Stage 3, No. (%)	26 (20.8)	34 (20.4)

KRT, No. (%)	22 (17.6)	23 (13.8)
Censoring events and competing risks		
Discharged alive from the ICU, No. (%)	571 (61.9)	477 (53.8)
Initiation of other treatment option, No. (%)	114 (12.4)	81 (9.1)
KRT without preceding AKI, No. (%)	2 (0.2)	9 (1.0)
Death in the ICU, No. (%)	43 (4.7)	49 (5.5)
Total ICU length of stay (Days), median (Q1 - Q3)	6.8 (3.8 - 12.9)	8.7 (4.9 - 16.8)
Total ICU mortality, No. (%)	122 (13.2)	147 (16.6)
Missing, %	0	0
Total hospital mortality, No. (%)	171 (18.5)	210 (23.7)
Missing, %	0	0

Back to table of contents

Supplement S8. Selected variables

Treatment models	Censoring models
Acute heart failure	Arm
Age	Acute heart failure
Alcohol abuse	Age
Ticagrelor	Alcohol abuse
Hydrochlorothiazide	Ticagrelor
Furosemide	Hydrochlorothiazide
Lisinopril	Furosemide
Cardiovascular disease	Lisinopril
Chronic kidney disease	Cardiovascular disease
Chronic pulmonary disease	Chronic kidney disease
SCr baseline	Chronic pulmonary disease
Diabetes mellitus	SCr baseline
eGFR	Diabetes mellitus
Sex	eGFR
Graft and transplant surgery	Sex
Hypoalbuminemia	Graft and transplant surgery
Hypotension	Hypoalbuminemia
Hypovolemia	Hypotension
Sulfamethoxazole and trimethoprim	Hypovolemia
Tobramycin	Sulfamethoxazole and trimethoprim
Gentamicin	Tobramycin
Amphotericin B	Gentamicin
Voriconazole	Amphotericin B
Aciclovir	Voriconazole
Ganciclovir	Aciclovir
Valaciclovir	Ganciclovir
Mycophenolic acid	Valaciclovir

Back to table of contents

Tacrolimus
Highest leukocyte count
Liver disease
Diclofenac
Baclofen
Major surgery
Malignancy
Lowest MAP
Mechanical ventilation
APACHE IV mortality probability
Obesity
Pre-initation SCr trend
Sepsis at admission
Sepsis
Lowest serum albumin
Highest SOFA score
Highest temperature
Time to initiation
Trauma
Lowest UO rate
ICU

Mycophenolic acid
Tacrolimus
Highest leukocyte count
Liver disease
Diclofenac
Baclofen
Major surgery
Malignancy
Lowest MAP
Mechanical ventilation
APACHE IV mortality probability
Obesity
Pre-initation SCr trend
Sepsis at admission
Lowest serum albumin
Highest SOFA score
Highest temperature
Time to initiation
Trauma
Lowest UO rate
ICU
Ticagrelor - longitudinal
Hydrochlorothiazide - longitudinal
Furosemide - longitudinal
Lisinopril - longitudinal
Hypoalbuminemia - longitudinal
Hypotension - longitudinal
Sulfamethoxazole and trimethoprim - longitudinal
Tobramycin - longitudinal
Gentamicin - longitudinal

Back to table of contents

Amphotericin B - longitudinal
Voriconazole - longitudinal
Aciclovir - longitudinal
Ganciclovir - longitudinal
Valaciclovir - longitudinal
Mycophenolic acid - longitudinal
Tacrolimus - longitudinal
Highest leukocyte count - longitudinal
Diclofenac - longitudinal
Baclofen - longitudinal
Lowest MAP - longitudinal
Sepsis - longitudinal
Lowest serum albumin - longitudinal
Highest SOFA score - longitudinal
Highest temperature - longitudinal
Lowest UO rate - longitudinal
Highest serum creatinine - longitudinal

Back to table of contents

Supplement S9. Overlap and balance assessment for IPTW – primary analysis

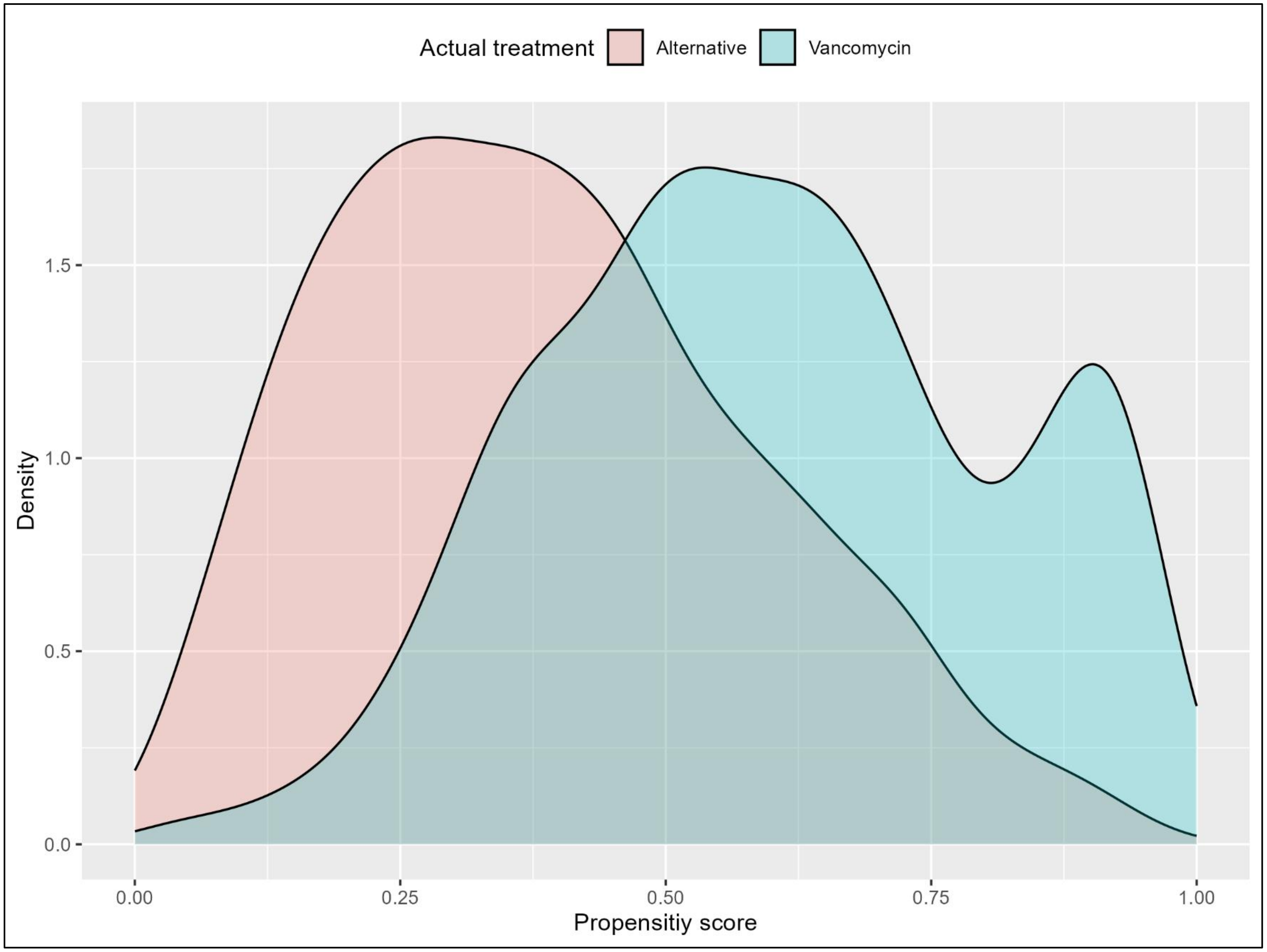

Supplementary Figure S9.1. Distributions of the predicted propensity scores for the vancomycin and alternative antibiotic treatment strategies.

Back to table of contents

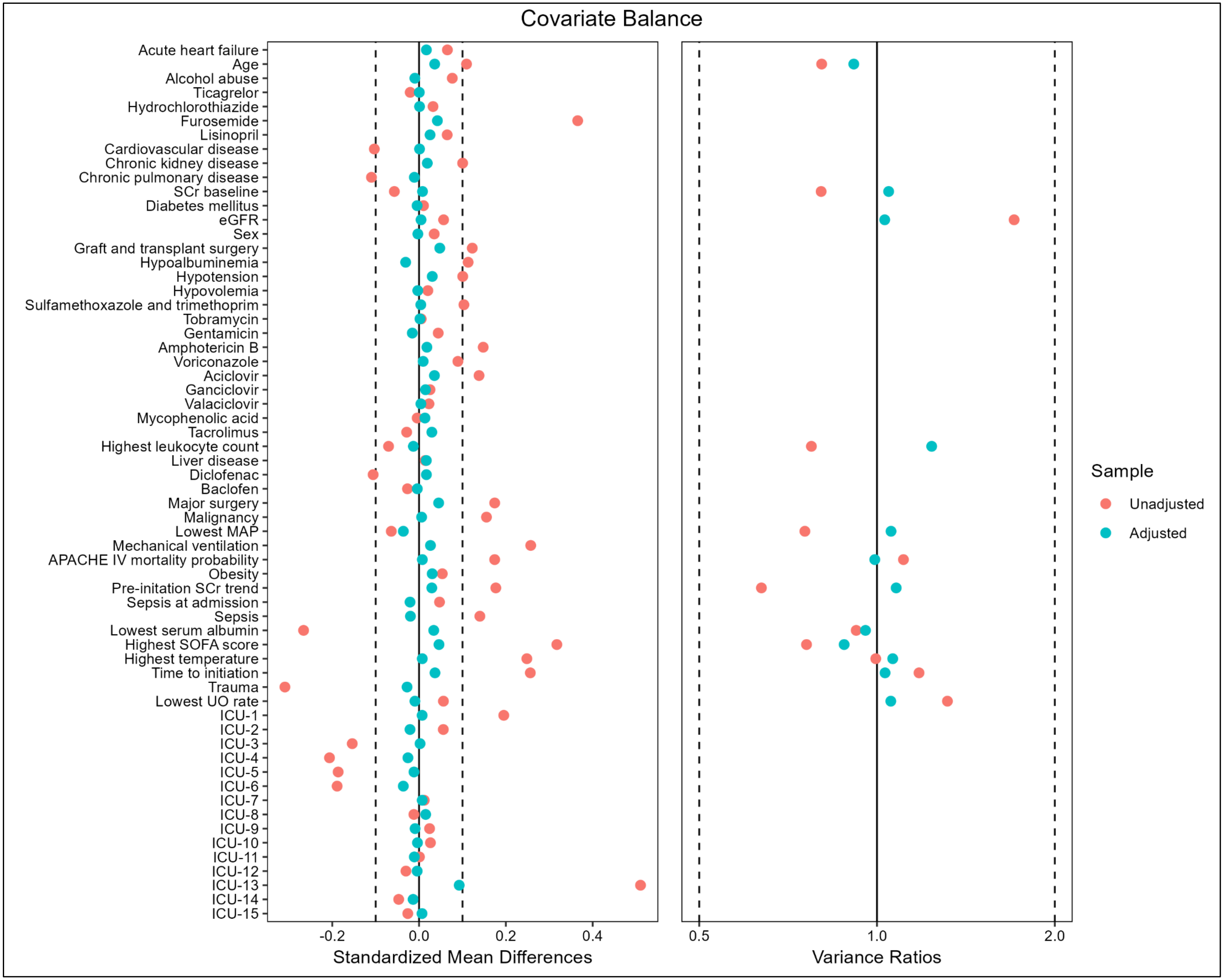

Supplementary Figure S9.2. Standardized mean differences and variance ratios for the potential confounders before and after IPTW.

Back to table of contents

Supplement S10. IPCW weights – primary analysis

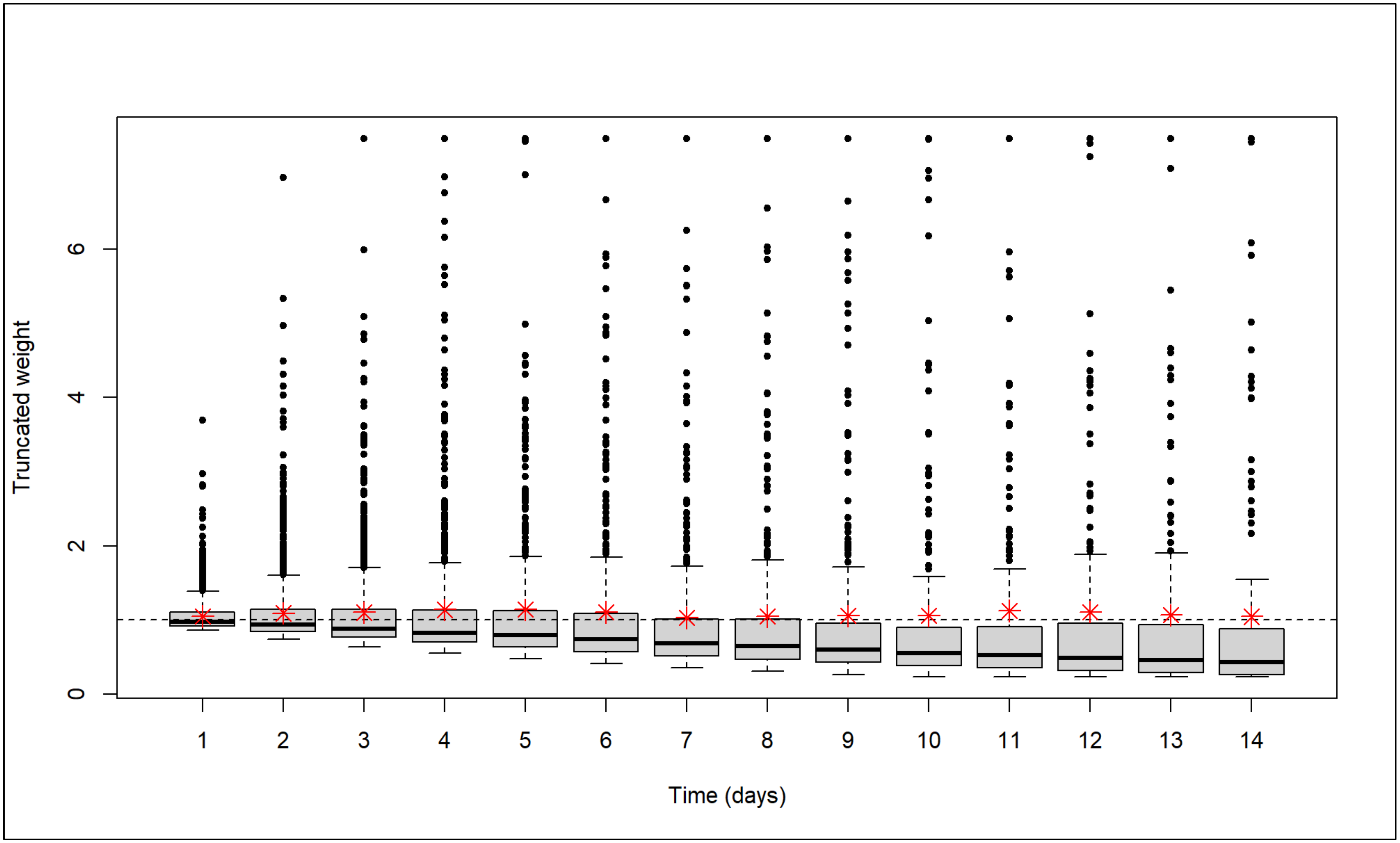

Supplementary Figure S10.1. IPCW weights for the primary analysis. The red stars represent the mean of the IPCW weights.

Back to table of contents

Supplement S11. Estimates – primary analysis

	Day	Risk Vancomcin	Risk Alternative	Risk Difference
Unadjusted	2	0.11 (0.07 - 0.14)	0.10 (0.08 - 0.12)	0.01 (-0.02 - 0.04)
IPTW	2	0.11 (0.07 - 0.14)	0.11 (0.09 - 0.13)	0.00 (-0.04 - 0.03)
IPCW	2	0.10 (0.06 - 0.12)	0.09 (0.07 - 0.10)	0.01 (-0.02 - 0.04)
IPTW+IPCW	2	0.10 (0.06 - 0.12)	0.10 (0.08 - 0.11)	0.00 (-0.03 - 0.03)
Unadjusted	14	0.29 (0.23 - 0.33)	0.24 (0.20 - 0.29)	0.05 (-0.03 - 0.10)
IPTW	14	0.31 (0.25 - 0.35)	0.25 (0.21 - 0.29)	0.06 (0.00 - 0.12)
IPCW	14	0.26 (0.19 - 0.31)	0.16 (0.14 - 0.20)	0.10 (0.01 - 0.16)
IPTW+IPCW	14	0.28 (0.21 - 0.34)	0.17 (0.14 - 0.20)	0.11 (0.04 - 0.19)

Back to table of contents

Supplement S12. Sensitivity analyses – primary analysis

Analysis	Adjustment	Day	Risk Difference
Main	IPTW+IPCW	14	0.11 (0.04 - 0.19)
Common support	IPTW+IPCW	14	0.11 (0.05 - 0.20)
No truncation	IPTW+IPCW	14	0.15 (0.05 - 0.22)
Effect not mediated by competing events	IPTW+IPCW	14	0.11 (0.03 - 0.17)
Multiple imputation	IPTW+IPCW	14	0.10 (0.03 - 0.18)
Main	IPTW	14	0.06 (0.00 - 0.12)
Separate censoring models	IPTW+IPCW (Discharge)	14	0.10 (0.03 - 0.16)
	IPTW+IPCW (Initiation of the other treatment strategy)	14	0.07 (0.01 - 0.13)

Back to table of contents

Supplement S13. Extended baseline characteristics and crude outcomes – exploratory analysis

Characteristic	Alternative (n = 652)	Vancomycin - lower dose (n = 322)	Vancomycin - higher dose (n = 354)
Arm options			
J01XA01 - Vancomycin, No. (%)	0 (0.0)	322 (100.0)	354 (100.0)
J01FF01 - Clindamycin, No. (%)	153 (23.5)	0 (0.0)	0 (0.0)
J01XX08 - Linezolid, No. (%)	15 (2.3)	0 (0.0)	0 (0.0)
J01XA02 - Teicoplanin, No. (%)	2 (0.3)	0 (0.0)	0 (0.0)
J01DH02 - Meropenem, No. (%)	246 (37.7)	0 (0.0)	0 (0.0)
J01DB04 - Cefazolin, No. (%)	235 (36.0)	0 (0.0)	0 (0.0)
J01XX09 - Daptomycin, No. (%)	1 (0.2)	0 (0.0)	0 (0.0)
Age (Years), median (Q1 - Q3)	63.0 (51.0 - 72.0)	65.0 (54.0 - 73.0)	65.0 (55.0 - 72.0)
Male sex, No. (%)	405 (62.1)	209 (64.9)	211 (59.6)
Planned admission, No. (%)	54 (8.3)	47 (14.6)	52 (14.7)
Admission type			
Medical, No. (%)	484 (74.3)	211 (65.5)	213 (60.2)
Emergency surgical, No. (%)	113 (17.4)	64 (19.9)	83 (23.4)
Elective surgical, No. (%)	54 (8.3)	47 (14.6)	58 (16.4)
APACHE IV score, median (Q1 - Q3)	68.0 (50.0 - 83.0)	70.0 (57.0 - 86.0)	70.5 (57.2 - 85.0)
APACHE IV mortality probability, median (Q1 - Q3)	0.2 (0.1 - 0.4)	0.3 (0.1 - 0.4)	0.3 (0.1 - 0.5)
SCr baseline (mg/dL), median (Q1 - Q3)	0.8 (0.7 - 1.2)	0.9 (0.7 - 1.2)	0.8 (0.6 - 1.1)
eGFR baseline (mL/min/1.73 m^2), median (Q1 - Q3)	83.2 (58.6 - 112.0)	81.2 (54.6 - 107.7)	82.9 (59.6 - 116.1)
Highest SCr during admission (mg/dL), median (Q1 - Q3)	0.9 (0.7 - 1.2)	1.0 (0.7 - 1.2)	0.9 (0.7 - 1.2)
SCr trend during admission (mg/dL per 3 days), median (Q1 - Q3)	-0.1 (-0.3 - 0.0)	-0.1 (-0.2 - 0.0)	-0.1 (-0.3 - 0.1)
Lowest urine output during admission (mL/kg/h), median (Q1 - Q3)	0.9 (0.7 - 1.3)	0.9 (0.7 - 1.2)	1.0 (0.7 - 1.4)
Lowest serum albumin during admission (g/dL), median (Q1 - Q3)	2.5 (2.1 - 2.7)	2.4 (1.8 - 2.6)	2.3 (1.7 - 2.5)
Lowest MAP during admission (mmHg), median (Q1 - Q3)	57.0 (51.0 - 63.0)	56.5 (50.0 - 63.0)	55.0 (48.2 - 61.0)
Highest SOFA score during admission, median (Q1 - Q3)	8.0 (5.0 - 10.0)	8.0 (7.0 - 10.0)	8.0 (6.0 - 10.0)
Highest temperature during admission (°C), median (Q1 - Q3)	38.4 (37.8 - 39.1)	38.7 (37.9 - 39.3)	38.6 (37.9 - 39.4)
Highest leukocyte count during admission (10**9 cells/L), median (Q1 - Q3)	16.6 (12.0 - 22.5)	15.9 (11.5 - 22.4)	18.2 (12.8 - 24.0)
Acute AKI risk factors			
Acute heart failure, No. (%)	74 (11.3)	43 (13.4)	42 (11.9)
Burns, No. (%)	1 (0.2)	0 (0.0)	0 (0.0)
Graft or transplant surgery, No. (%)	35 (5.4)	19 (5.9)	26 (7.3)
Hypoalbuminemia, No. (%)	541 (83.0)	281 (87.3)	312 (88.1)
Hypotension, No. (%)	206 (31.6)	122 (37.9)	139 (39.3)
Hypovolemia, No. (%)	16 (2.5)	10 (3.1)	11 (3.1)
Major surgery, No. (%)	147 (22.5)	88 (27.3)	119 (33.6)
Mechanical ventilation, No. (%)	445 (68.3)	247 (76.7)	274 (77.4)
Sepsis - admission diagnosis, No. (%)	101 (15.5)	58 (18.0)	54 (15.3)
Sepsis - longitudinal, No. (%)	240 (36.8)	141 (43.8)	154 (43.5)

Back to table of contents

Trauma, No. (%)	78 (12.0)	10 (3.1)	9 (2.5)
Chronic AKI risk factors			
Alcohol abuse, No. (%)	139 (21.3)	78 (24.2)	93 (26.3)
Cardiovascular disease, No. (%)	184 (28.2)	69 (21.4)	96 (27.1)
Chronic kidney disease, No. (%)	8 (1.2)	10 (3.1)	5 (1.4)
Chronic pulmonary disease, No. (%)	108 (16.6)	48 (14.9)	42 (11.9)
Diabetes mellitus, No. (%)	87 (13.3)	58 (18.0)	43 (12.1)
Liver disease, No. (%)	9 (1.4)	4 (1.2)	4 (1.1)
Malignancy, No. (%)	74 (11.3)	59 (18.3)	66 (18.6)
Obesity, No. (%)	92 (14.1)	77 (23.9)	34 (9.6)
APACHE IV admission diagnosis category			
Cardiovascular, No. (%)	161 (24.7)	99 (30.7)	107 (30.2)
Gastrointestinal, No. (%)	103 (15.8)	85 (26.4)	107 (30.2)
Genitourinary, No. (%)	4 (0.6)	4 (1.2)	5 (1.4)
Hematology, No. (%)	4 (0.6)	5 (1.6)	3 (0.8)
Metabolic/Endocrine, No. (%)	12 (1.8)	4 (1.2)	3 (0.8)
Musculoskeletal/Skin, No. (%)	7 (1.1)	2 (0.6)	3 (0.8)
Neurologic, No. (%)	78 (12.0)	29 (9.0)	25 (7.1)
Respiratory, No. (%)	200 (30.7)	83 (25.8)	94 (26.6)
Transplant, No. (%)	9 (1.4)	1 (0.3)	0 (0.0)
Trauma, No. (%)	74 (11.3)	10 (3.1)	7 (2.0)
Nephrotoxin exposure			
A07EC01 - sulfasalazine, No. (%)	0 (0.0)	1 (0.3)	0 (0.0)
A07EC02 - mesalazine, No. (%)	0 (0.0)	0 (0.0)	3 (0.8)
B01AC24 - ticagrelor, No. (%)	13 (2.0)	6 (1.9)	8 (2.3)
C03AA03 - hydrochlorothiazide, No. (%)	19 (2.9)	10 (3.1)	13 (3.7)
C03CA01 - furosemide, No. (%)	249 (38.2)	197 (61.2)	175 (49.4)
C07AG02 - carvedilol, No. (%)	0 (0.0)	0 (0.0)	2 (0.6)
C09AA02 - enalapril, No. (%)	7 (1.1)	4 (1.2)	2 (0.6)
C09AA03 - lisinopril, No. (%)	5 (0.8)	3 (0.9)	7 (2.0)
C09CA01 - losartan, No. (%)	3 (0.5)	1 (0.3)	3 (0.8)
C09CA03 - valsartan, No. (%)	2 (0.3)	2 (0.6)	0 (0.0)
C09CA06 - candesartan, No. (%)	1 (0.2)	0 (0.0)	0 (0.0)
J01EE01 - sulfamethoxazole and trimethoprim, No. (%)	27 (4.1)	23 (7.1)	21 (5.9)
J01GB01 - tobramycin, No. (%)	8 (1.2)	1 (0.3)	6 (1.7)
J01GB03 - gentamicin, No. (%)	89 (13.7)	37 (11.5)	60 (16.9)
J01GB06 - amikacin, No. (%)	0 (0.0)	1 (0.3)	0 (0.0)
J01XB01 - colistin, No. (%)	5 (0.8)	0 (0.0)	0 (0.0)
J02AA01 - amphotericin B, No. (%)	5 (0.8)	6 (1.9)	12 (3.4)
J02AC03 - voriconazole, No. (%)	7 (1.1)	5 (1.6)	11 (3.1)
J02AX04 - caspofungin, No. (%)	4 (0.6)	6 (1.9)	5 (1.4)

Back to table of contents

J02AX06 - anidulafungin, No. (%)	2 (0.3)	5 (1.6)	4 (1.1)
J05AB01 - aciclovir, No. (%)	8 (1.2)	11 (3.4)	10 (2.8)
J05AB06 - ganciclovir, No. (%)	6 (0.9)	2 (0.6)	6 (1.7)
J05AB11 - valaciclovir, No. (%)	10 (1.5)	3 (0.9)	11 (3.1)
J05AB14 - valganciclovir, No. (%)	1 (0.2)	0 (0.0)	0 (0.0)
J05AD01 - foscarnet, No. (%)	0 (0.0)	0 (0.0)	1 (0.3)
J05AE03 - ritonavir, No. (%)	0 (0.0)	0 (0.0)	0 (0.0)
L01AA01 - cyclophosphamide, No. (%)	0 (0.0)	1 (0.3)	0 (0.0)
L01BC01 - cytarabine, No. (%)	0 (0.0)	0 (0.0)	0 (0.0)
L01XA01 - cisplatin, No. (%)	0 (0.0)	1 (0.3)	0 (0.0)
L01XX05 - hydroxycarbamide, No. (%)	0 (0.0)	2 (0.6)	0 (0.0)
L03AA02 - filgrastim, No. (%)	2 (0.3)	5 (1.6)	7 (2.0)
L04AA06 - mycophenolic acid, No. (%)	11 (1.7)	7 (2.2)	3 (0.8)
L04AA18 - everolimus, No. (%)	0 (0.0)	0 (0.0)	0 (0.0)
L04AC02 - basiliximab, No. (%)	5 (0.8)	0 (0.0)	1 (0.3)
L04AD01 - ciclosporin, No. (%)	2 (0.3)	2 (0.6)	3 (0.8)
L04AD02 - tacrolimus, No. (%)	9 (1.4)	4 (1.2)	2 (0.6)
M01AB05 - diclofenac, No. (%)	19 (2.9)	4 (1.2)	5 (1.4)
M01AE01 - ibuprofen, No. (%)	1 (0.2)	0 (0.0)	0 (0.0)
M01AE02 - naproxen, No. (%)	1 (0.2)	0 (0.0)	0 (0.0)
M01AH01 - celecoxib, No. (%)	0 (0.0)	0 (0.0)	0 (0.0)
M03BX01 - baclofen, No. (%)	10 (1.5)	5 (1.6)	3 (0.8)
M04AC01 - colchicine, No. (%)	0 (0.0)	1 (0.3)	1 (0.3)
M05BA03 - pamidronic acid, No. (%)	1 (0.2)	1 (0.3)	0 (0.0)
N05AH02 - clozapine, No. (%)	2 (0.3)	4 (1.2)	0 (0.0)
N05AN01 - lithium, No. (%)	3 (0.5)	2 (0.6)	0 (0.0)
Time in ICU before initiation (Days), median (Q1 - Q3)	2.1 (1.5 - 3.8)	2.9 (1.7 - 4.8)	2.7 (1.8 - 4.0)
AKI, No. (%)	67 (10.3)	60 (18.6)	49 (13.8)
AKI stage			
Stage 1, No. (%)	44 (65.7)	40 (66.7)	29 (59.2)
Stage 2, No. (%)	8 (11.9)	10 (16.7)	10 (20.4)
Stage 3, No. (%)	15 (22.4)	10 (16.7)	10 (20.4)
KRT, No. (%)	13 (19.4)	5 (8.3)	6 (12.2)
Censoring events and competing risks			
Discharged alive from the ICU, No. (%)	419 (64.3)	185 (57.5)	193 (54.5)
Initiation of other treatment option, No. (%)	68 (10.4)	14 (4.3)	28 (7.9)
KRT without preceding AKI, No. (%)	0 (0.0)	3 (0.9)	0 (0.0)
Death in the ICU, No. (%)	31 (4.8)	12 (3.7)	28 (7.9)
Total ICU length of stay (Days), median (Q1 - Q3)	8.3 (5.0 - 15.2)	10.2 (6.1 - 18.9)	9.4 (5.8 - 17.3)
Total ICU mortality, No. (%)	73 (11.2)	42 (13.0)	62 (17.5)
Total hospital mortality, No. (%)	105 (16.1)	69 (21.4)	88 (24.9)

Back to table of contents

Supplement S14. Extended baseline characteristics and crude outcomes with missing data – exploratory analysis

Characteristic	Alternative (n = 652)	Vancomycin - lower dose (n = 322)	Vancomycin - higher dose (n = 354)
Arm options			
J01XA01 - Vancomycin, No. (%)	0 (0.0)	322 (100.0)	354 (100.0)
J01FF01 - Clindamycin, No. (%)	153 (23.5)	0 (0.0)	0 (0.0)
J01XX08 - Linezolid, No. (%)	15 (2.3)	0 (0.0)	0 (0.0)
J01XA02 - Teicoplanin, No. (%)	2 (0.3)	0 (0.0)	0 (0.0)
J01DH02 - Meropenem, No. (%)	246 (37.7)	0 (0.0)	0 (0.0)
J01DB04 - Cefazolin, No. (%)	235 (36.0)	0 (0.0)	0 (0.0)
J01XX09 - Daptomycin, No. (%)	1 (0.2)	0 (0.0)	0 (0.0)
Age (Years), median (Q1 - Q3)	63.0 (51.0 - 72.0)	65.0 (54.0 - 73.0)	65.0 (55.0 - 72.0)
Missing, %	0	0	0
Male sex, No. (%)	405 (62.1)	209 (64.9)	211 (59.6)
Missing, %	0	0	0
Planned admission, No. (%)	54 (8.3)	47 (14.6)	52 (14.7)
Missing, %	0	0	0
Admission type			
Medical, No. (%)	484 (74.3)	211 (65.5)	213 (60.2)
Emergency surgical, No. (%)	113 (17.4)	64 (19.9)	83 (23.4)
Elective surgical, No. (%)	54 (8.3)	47 (14.6)	58 (16.4)
Missing, %	0	0	0
APACHE IV score, median (Q1 - Q3)	68.0 (50.0 - 83.0)	70.0 (57.0 - 86.0)	70.5 (57.2 - 85.0)
Missing, %	0	0	0
APACHE IV mortality probability, median (Q1 - Q3)	0.2 (0.1 - 0.4)	0.2 (0.1 - 0.4)	0.3 (0.1 - 0.5)
Missing, %	1	1	2
SCr baseline (mg/dL), median (Q1 - Q3)	0.8 (0.7 - 1.2)	0.9 (0.7 - 1.2)	0.8 (0.6 - 1.1)
Missing, %	0	0	0
eGFR baseline (mL/min/1.73 m²), median (Q1 - Q3)	83.2 (58.6 - 112.0)	81.2 (54.6 - 107.7)	82.9 (59.6 - 116.1)
Missing, %	0	0	0
Highest SCr during admission (mg/dL), median (Q1 - Q3)	0.9 (0.7 - 1.2)	1.0 (0.7 - 1.2)	0.9 (0.7 - 1.2)
Missing, %	0	0	0
SCr trend during admission (mg/dL per 3 days), median (Q1 - Q3)	-0.1 (-0.3 - 0.0)	-0.1 (-0.2 - 0.0)	-0.1 (-0.3 - 0.1)
Missing, %	4	3	3
Lowest urine output during admission (mL/kg/h), median (Q1 - Q3)	0.9 (0.7 - 1.3)	0.9 (0.7 - 1.2)	1.0 (0.7 - 1.4)
Missing, %	3	2	1
Lowest serum albumin during admission (g/dL), median (Q1 - Q3)	2.4 (1.9 - 2.9)	2.2 (1.7 - 2.8)	2.2 (1.7 - 2.7)
Missing, %	22	16	12
Lowest MAP during admission (mmHg), median (Q1 - Q3)	57.0 (51.0 - 63.0)	56.3 (50.0 - 63.0)	55.0 (48.2 - 61.0)
Missing, %	1	0	0
Highest SOFA score during admission, median (Q1 - Q3)	7.0 (5.0 - 10.0)	8.0 (7.0 - 10.0)	8.0 (6.0 - 10.0)

Back to table of contents

Missing, %	0	0	0
Highest temperature during admission (°C), median (Q1 - Q3)	38.4 (37.8 - 39.1)	38.7 (37.9 - 39.3)	38.6 (37.9 - 39.4)
Missing, %	1	1	0
Highest leukocyte count during admission (10**9 cells/L), median (Q1 - Q3)	16.5 (12.0 - 22.6)	15.9 (11.4 - 22.4)	18.3 (12.7 - 24.1)
Missing, %	1	1	1
Acute AKI risk factors			
Acute heart failure, No. (%)	74 (11.3)	43 (13.4)	42 (11.9)
Missing, %	0	0	0
Burns, No. (%)	1 (0.2)	0 (0.0)	0 (0.0)
Missing, %	0	0	0
Graft or transplant surgery, No. (%)	35 (5.4)	19 (5.9)	26 (7.3)
Missing, %	0	0	0
Hypoalbuminemia, No. (%)	396 (60.7)	226 (70.2)	267 (75.4)
Missing, %	22	16	12
Hypotension, No. (%)	206 (31.6)	122 (37.9)	139 (39.3)
Missing, %	1	0	0
Hypovolemia, No. (%)	16 (2.5)	10 (3.1)	11 (3.1)
Missing, %	0	0	0
Major surgery, No. (%)	147 (22.5)	88 (27.3)	119 (33.6)
Missing, %	0	0	0
Mechanical ventilation, No. (%)	445 (68.3)	247 (76.7)	274 (77.4)
Missing, %	0	0	0
Sepsis - admission diagnosis, No. (%)	101 (15.5)	58 (18.0)	54 (15.3)
Missing, %	0	0	0
Sepsis - longitudinal, No. (%)	240 (36.8)	141 (43.8)	154 (43.5)
Missing, %	0	0	0
Trauma, No. (%)	78 (12.0)	10 (3.1)	9 (2.5)
Missing, %	0	0	0
Chronic AKI risk factors			
Alcohol abuse, No. (%)	139 (21.3)	78 (24.2)	93 (26.3)
Missing, %	0	0	0
Cardiovascular disease, No. (%)	184 (28.2)	69 (21.4)	96 (27.1)
Missing, %	0	0	0
Chronic kidney disease, No. (%)	8 (1.2)	10 (3.1)	5 (1.4)
Missing, %	0	0	0
Chronic pulmonary disease, No. (%)	108 (16.6)	48 (14.9)	42 (11.9)
Missing, %	0	0	0
Diabetes mellitus, No. (%)	87 (13.3)	58 (18.0)	43 (12.1)
Missing, %	0	0	0
Liver disease, No. (%)	9 (1.4)	4 (1.2)	4 (1.1)
Missing, %	0	0	0

Back to table of contents

Malignancy, No. (%)	74 (11.3)	59 (18.3)	66 (18.6)
Missing, %	0	0	0
Obesity, No. (%)	92 (14.5)	77 (24.5)	34 (9.8)
Missing, %	2	2	2
APACHE IV admission diagnosis category			
Cardiovascular, No. (%)	161 (24.7)	99 (30.7)	107 (30.2)
Gastrointestinal, No. (%)	103 (15.8)	85 (26.4)	107 (30.2)
Genitourinary, No. (%)	4 (0.6)	4 (1.2)	5 (1.4)
Hematology, No. (%)	4 (0.6)	5 (1.6)	3 (0.8)
Metabolic/Endocrine, No. (%)	12 (1.8)	4 (1.2)	3 (0.8)
Musculoskeletal/Skin, No. (%)	7 (1.1)	2 (0.6)	3 (0.8)
Neurologic, No. (%)	78 (12.0)	29 (9.0)	25 (7.1)
Respiratory, No. (%)	200 (30.7)	83 (25.8)	94 (26.6)
Transplant, No. (%)	9 (1.4)	1 (0.3)	0 (0.0)
Trauma, No. (%)	74 (11.3)	10 (3.1)	7 (2.0)
Missing, %	0	0	0
Nephrotoxin exposure			
A07EC01 - sulfasalazine, No. (%)	0 (0.0)	1 (0.3)	0 (0.0)
A07EC02 - mesalazine, No. (%)	0 (0.0)	0 (0.0)	3 (0.8)
B01AC24 - ticagrelor, No. (%)	13 (2.0)	6 (1.9)	8 (2.3)
C03AA03 - hydrochlorothiazide, No. (%)	19 (2.9)	10 (3.1)	13 (3.7)
C03CA01 - furosemide, No. (%)	249 (38.2)	197 (61.2)	175 (49.4)
C07AG02 - carvedilol, No. (%)	0 (0.0)	0 (0.0)	2 (0.6)
C09AA02 - enalapril, No. (%)	7 (1.1)	4 (1.2)	2 (0.6)
C09AA03 - lisinopril, No. (%)	5 (0.8)	3 (0.9)	7 (2.0)
C09CA01 - losartan, No. (%)	3 (0.5)	1 (0.3)	3 (0.8)
C09CA03 - valsartan, No. (%)	2 (0.3)	2 (0.6)	0 (0.0)
C09CA06 - candesartan, No. (%)	1 (0.2)	0 (0.0)	0 (0.0)
J01EE01 - sulfamethoxazole and trimethoprim, No. (%)	27 (4.1)	23 (7.1)	21 (5.9)
J01GB01 - tobramycin, No. (%)	8 (1.2)	1 (0.3)	6 (1.7)
J01GB03 - gentamicin, No. (%)	89 (13.7)	37 (11.5)	60 (16.9)
J01GB06 - amikacin, No. (%)	0 (0.0)	1 (0.3)	0 (0.0)
J01XB01 - colistin, No. (%)	5 (0.8)	0 (0.0)	0 (0.0)
J02AA01 - amphotericin B, No. (%)	5 (0.8)	6 (1.9)	12 (3.4)
J02AC03 - voriconazole, No. (%)	7 (1.1)	5 (1.6)	11 (3.1)
J02AX04 - caspofungin, No. (%)	4 (0.6)	6 (1.9)	5 (1.4)
J02AX06 - anidulafungin, No. (%)	2 (0.3)	5 (1.6)	4 (1.1)
J05AB01 - aciclovir, No. (%)	8 (1.2)	11 (3.4)	10 (2.8)
J05AB06 - ganciclovir, No. (%)	6 (0.9)	2 (0.6)	6 (1.7)
J05AB11 - valaciclovir, No. (%)	10 (1.5)	3 (0.9)	11 (3.1)
J05AB14 - valganciclovir, No. (%)	1 (0.2)	0 (0.0)	0 (0.0)

Back to table of contents

J05AD01 - foscarnet, No. (%)	0 (0.0)	0 (0.0)	1 (0.3)
J05AE03 - ritonavir, No. (%)	0 (0.0)	0 (0.0)	0 (0.0)
L01AA01 - cyclophosphamide, No. (%)	0 (0.0)	1 (0.3)	0 (0.0)
L01BC01 - cytarabine, No. (%)	0 (0.0)	0 (0.0)	0 (0.0)
L01XA01 - cisplatin, No. (%)	0 (0.0)	1 (0.3)	0 (0.0)
L01XX05 - hydroxycarbamide, No. (%)	0 (0.0)	2 (0.6)	0 (0.0)
L03AA02 - filgrastim, No. (%)	2 (0.3)	5 (1.6)	7 (2.0)
L04AA06 - mycophenolic acid, No. (%)	11 (1.7)	7 (2.2)	3 (0.8)
L04AA18 - everolimus, No. (%)	0 (0.0)	0 (0.0)	0 (0.0)
L04AC02 - basiliximab, No. (%)	5 (0.8)	0 (0.0)	1 (0.3)
L04AD01 - ciclosporin, No. (%)	2 (0.3)	2 (0.6)	3 (0.8)
L04AD02 - tacrolimus, No. (%)	9 (1.4)	4 (1.2)	2 (0.6)
M01AB05 - diclofenac, No. (%)	19 (2.9)	4 (1.2)	5 (1.4)
M01AE01 - ibuprofen, No. (%)	1 (0.2)	0 (0.0)	0 (0.0)
M01AE02 - naproxen, No. (%)	1 (0.2)	0 (0.0)	0 (0.0)
M01AH01 - celecoxib, No. (%)	0 (0.0)	0 (0.0)	0 (0.0)
M03BX01 - baclofen, No. (%)	10 (1.5)	5 (1.6)	3 (0.8)
M04AC01 - colchicine, No. (%)	0 (0.0)	1 (0.3)	1 (0.3)
M05BA03 - pamidronic acid, No. (%)	1 (0.2)	1 (0.3)	0 (0.0)
N05AH02 - clozapine, No. (%)	2 (0.3)	4 (1.2)	0 (0.0)
N05AN01 - lithium, No. (%)	3 (0.5)	2 (0.6)	0 (0.0)
Time in ICU before initiation (Days), median (Q1 - Q3)	2.1 (1.5 - 3.8)	2.9 (1.7 - 4.8)	2.7 (1.8 - 4.0)
AKI, No. (%)	67 (10.3)	60 (18.6)	49 (13.8)
AKI stage			
Stage 1, No. (%)	44 (65.7)	40 (66.7)	29 (59.2)
Stage 2, No. (%)	8 (11.9)	10 (16.7)	10 (20.4)
Stage 3, No. (%)	15 (22.4)	10 (16.7)	10 (20.4)
KRT, No. (%)	13 (19.4)	5 (8.3)	6 (12.2)
Censoring events and competing risks			
Discharged alive from the ICU, No. (%)	419 (64.3)	185 (57.5)	193 (54.5)
Initiation of other treatment option, No. (%)	68 (10.4)	14 (4.3)	28 (7.9)
KRT without preceding AKI, No. (%)	0 (0.0)	3 (0.9)	0 (0.0)
Death in the ICU, No. (%)	31 (4.8)	12 (3.7)	28 (7.9)
Total ICU length of stay (Days), median (Q1 - Q3)	8.3 (5.0 - 15.2)	10.2 (6.1 - 18.9)	9.4 (5.8 - 17.3)
Total ICU mortality, No. (%)	73 (11.2)	42 (13.0)	62 (17.5)
Missing, %	0	0	0
Total hospital mortality, No. (%)	105 (16.1)	69 (21.4)	88 (24.9)
Missing, %	0	0	0

Back to table of contents

Supplement S15. Overlap and balance assessments for IPTW – exploratory analysis

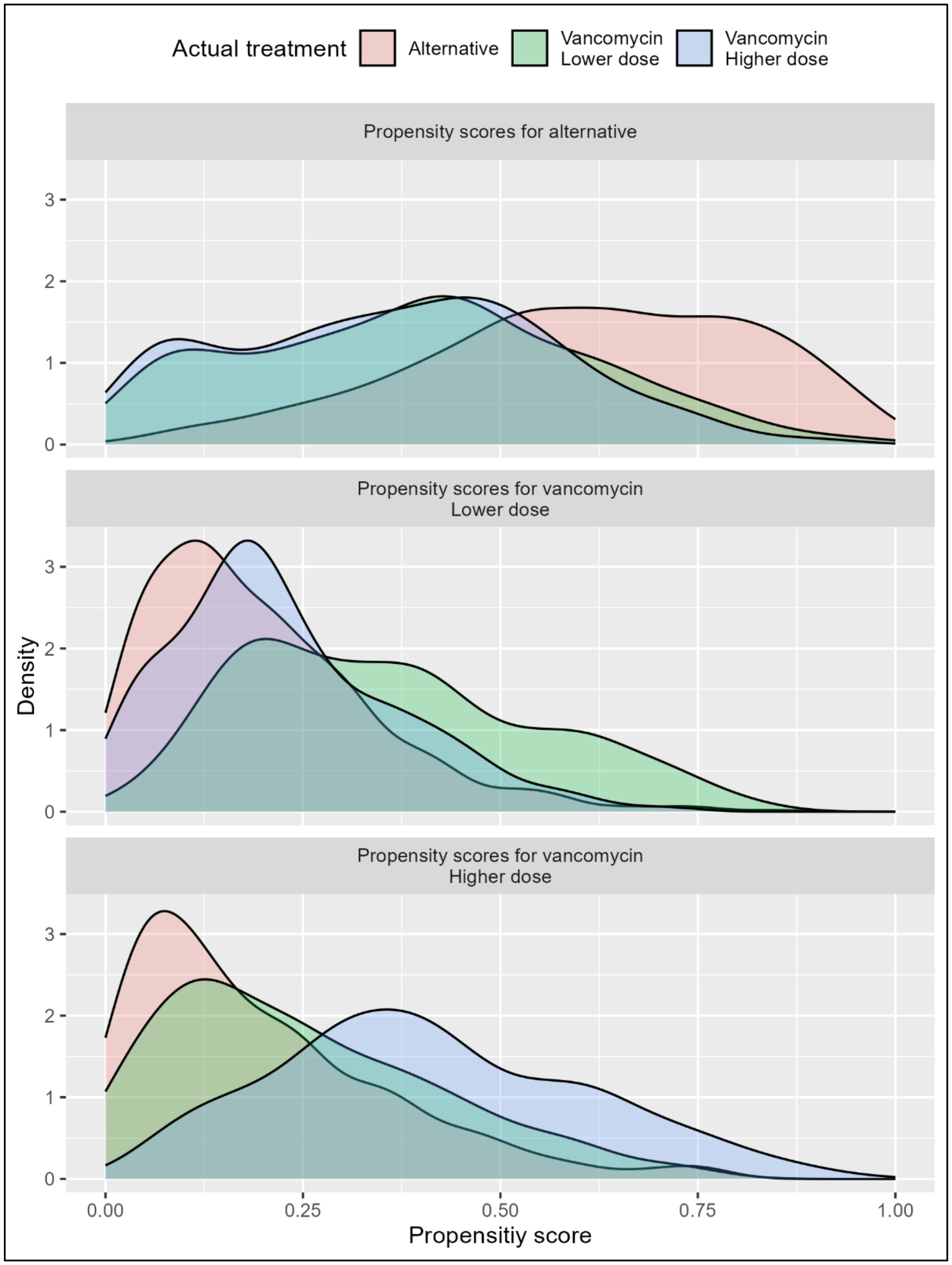

Supplementary Figure S15.1. Distributions of the predicted propensity scores for the three treatment strategies.

Back to table of contents

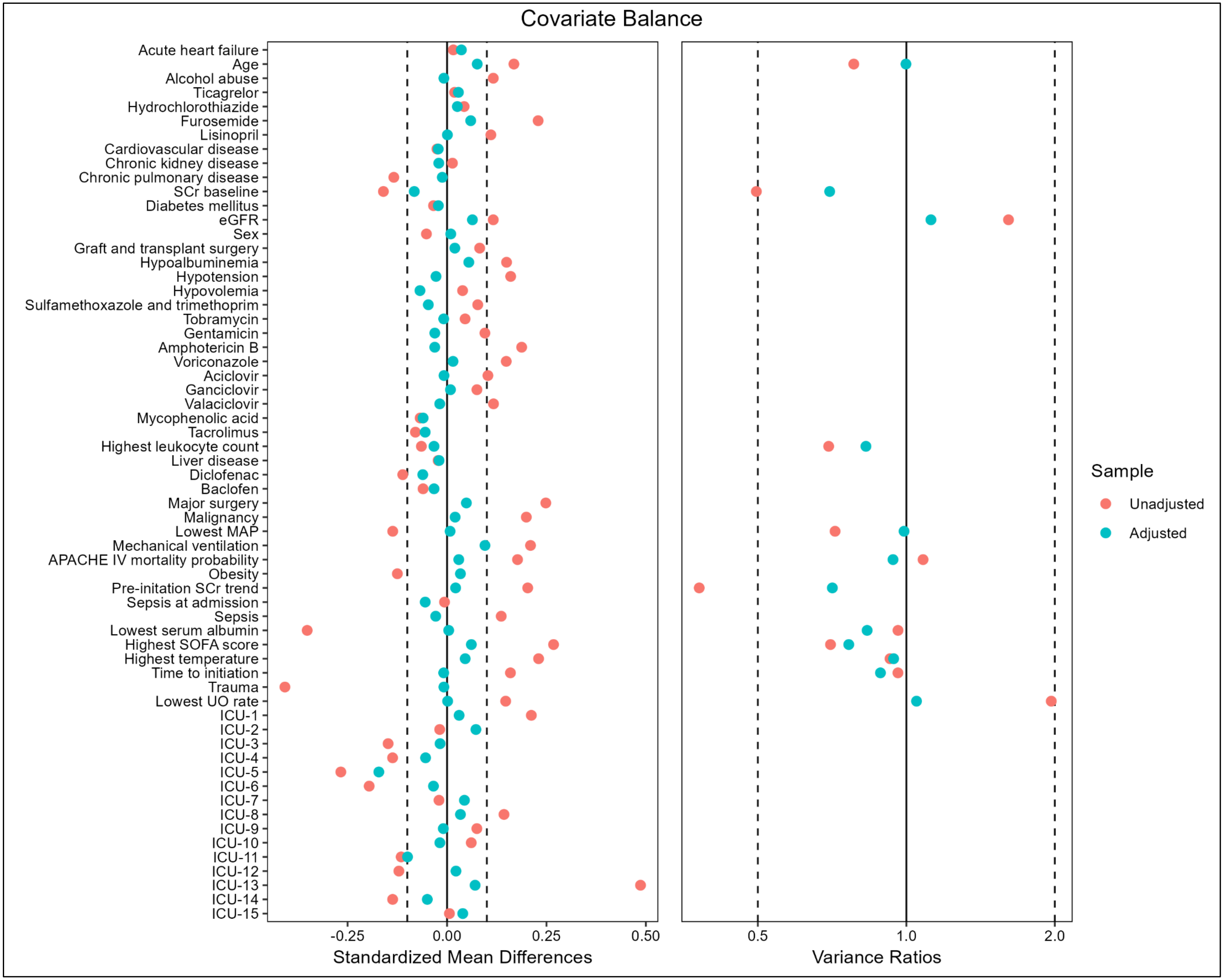

Supplementary Figure S15.2. Standardized mean differences and variance ratios for the potential confounders before and after IPTW in the comparison between the higher vancomycin dose and the alternative antibiotic treatment strategies.

Back to table of contents

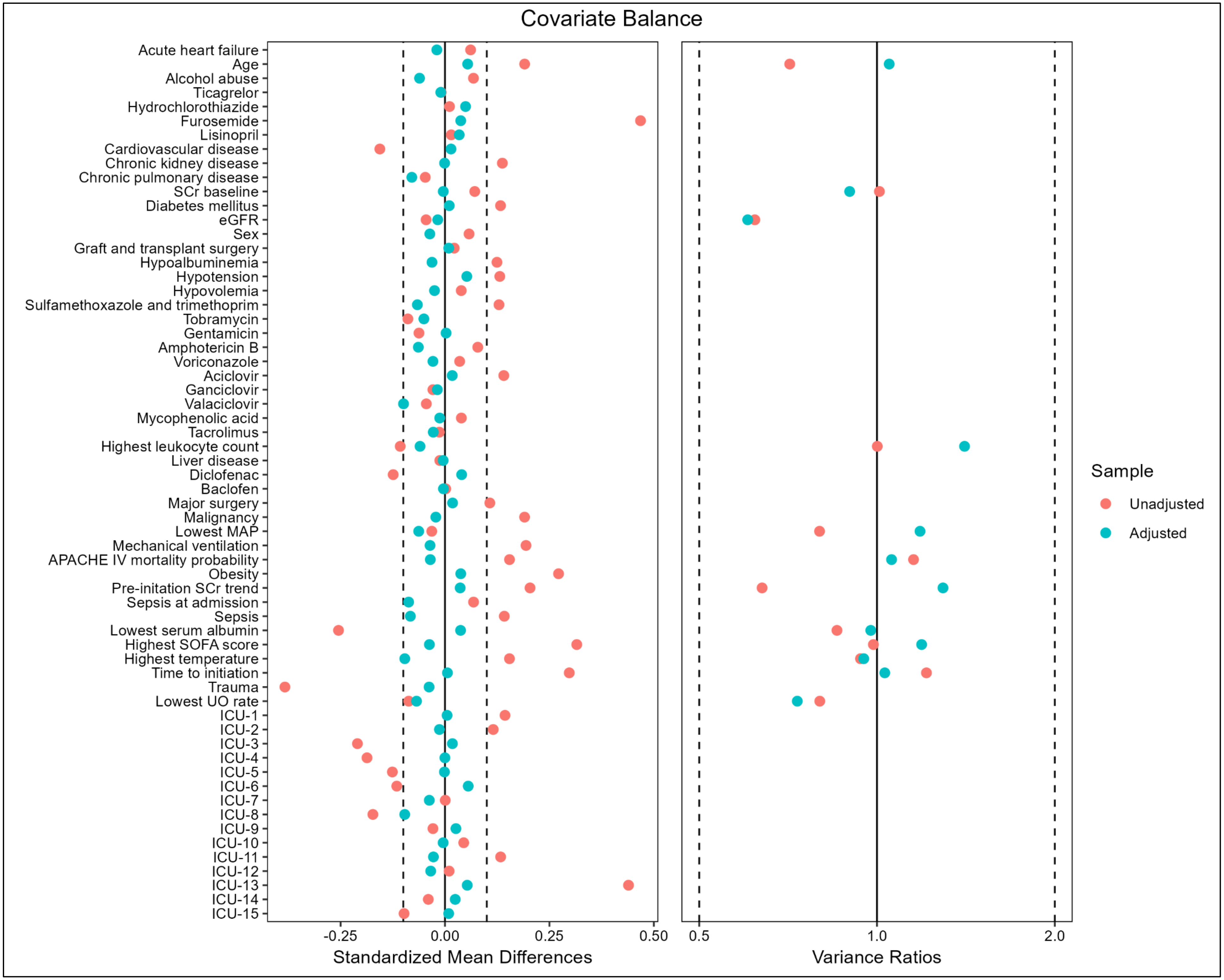

Supplementary Figure S15.3. Standardized mean differences and variance ratios for the potential confounders before and after IPTW in the comparison between the lower vancomycin dose and the alternative antibiotic treatment strategies.

Back to table of contents

Supplement S16. IPCW weights – exploratory analysis

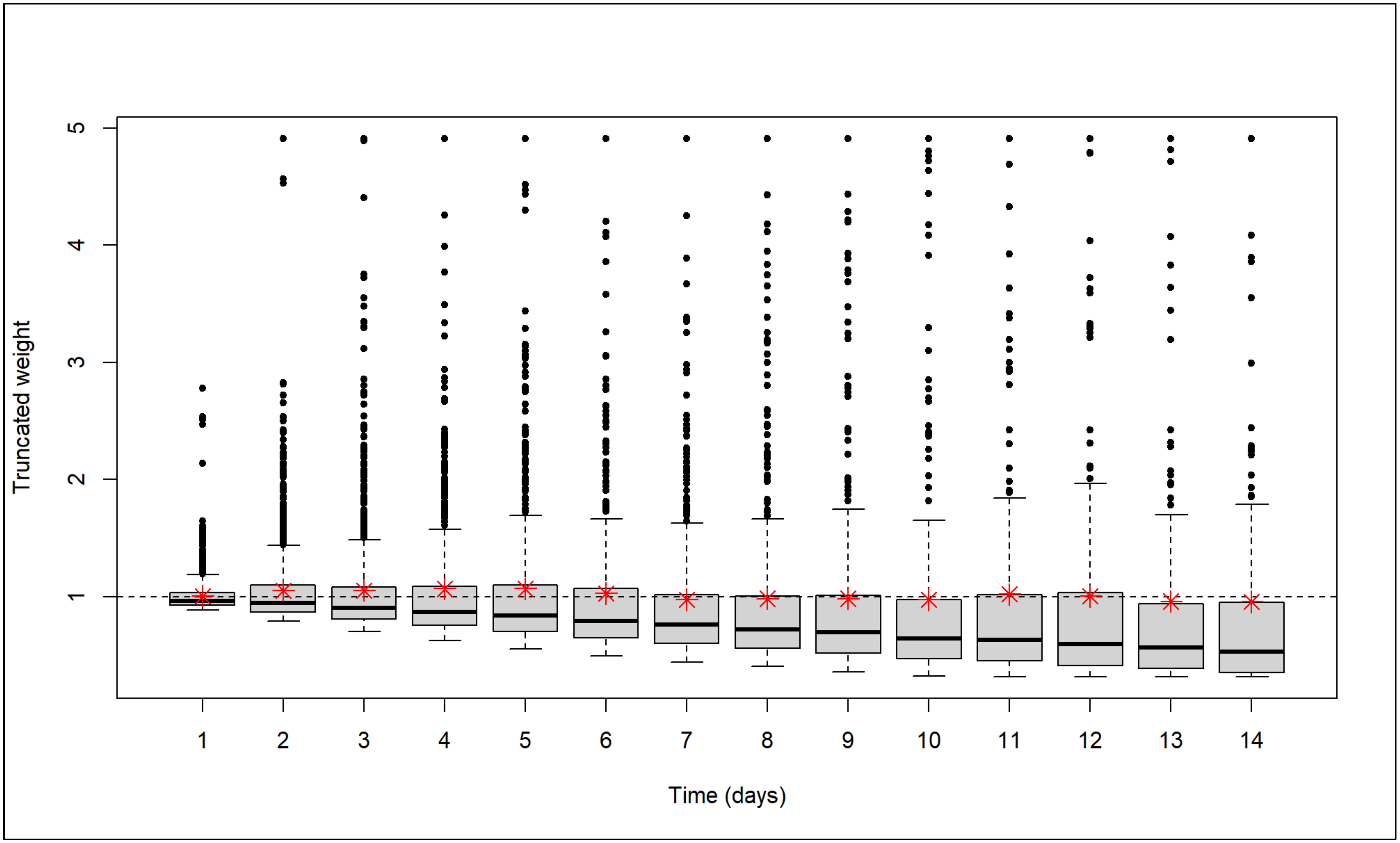

Supplementary Figure S16.1. IPCW weights for the exploratory analysis. The red stars represent the mean of the IPCW weights.

Back to table of contents

Supplement S17. Estimates – exploratory analysis

	Day	Risk Vancomycin Higher dose	Risk Vancomycin Lower dose	Risk Alternative	Risk difference Vancomycin Higher dose vs Alternative	Risk difference Vancomycin Lower dose vs Alternative
Unadjusted	2	0.04 (0.02 - 0.06)	0.05 (0.03 - 0.07)	0.03 (0.02 - 0.05)	0.01 (-0.01 - 0.03)	0.02 (-0.01 - 0.03)
IPTW	2	0.05 (0.02 - 0.08)	0.04 (0.02 - 0.06)	0.06 (0.03 - 0.08)	-0.01 (-0.04 - 0.02)	-0.02 (-0.04 - 0.01)
IPCW	2	0.04 (0.01 - 0.05)	0.04 (0.02 - 0.06)	0.02 (0.01 - 0.03)	0.01 (-0.01 - 0.02)	0.02 (0.00 - 0.03)
IPTW+IPCW	2	0.04 (0.01 - 0.07)	0.03 (0.01 - 0.05)	0.04 (0.02 - 0.06)	0.00 (-0.03 - 0.02)	-0.01 (-0.03 - 0.02)
Unadjusted	14	0.22 (0.17 - 0.25)	0.27 (0.20 - 0.32)	0.19 (0.14 - 0.24)	0.03 (-0.05 - 0.09)	0.08 (0.00 - 0.14)
IPTW	14	0.30 (0.20 - 0.34)	0.29 (0.20 - 0.39)	0.21 (0.16 - 0.25)	0.09 (-0.01 - 0.13)	0.08 (-0.01 - 0.19)
IPCW	14	0.22 (0.15 - 0.28)	0.21 (0.15 - 0.25)	0.12 (0.09 - 0.15)	0.10 (0.01 - 0.17)	0.09 (0.01 - 0.13)
IPTW+IPCW	14	0.30 (0.19 - 0.37)	0.23 (0.15 - 0.32)	0.13 (0.10 - 0.16)	0.17 (0.05 - 0.25)	0.10 (0.01 - 0.19)

Back to table of contents

Supplement S18. Sensitivity analyses – exploratory analysis

Analysis	Adjustment	Day	Risk difference Vancomycin Higher dose vs Alternative	Risk difference Vancomycin Lower dose vs Alternative
Main	IPTW+IPCW	14	0.17 (0.05 - 0.25)	0.10 (0.01 - 0.19)
Common support	IPTW+IPCW	14	0.19 (0.08 - 0.30)	0.14 (0.04 - 0.26)
No landmark	IPTW+IPCW	14	0.20 (0.10 - 0.32)	0.07 (-0.02 - 0.17)
No truncation	IPTW+IPCW	14	0.19 (0.05 - 0.27)	0.12 (0.02 - 0.24)
Effect not mediated by competing events	IPTW+IPCW	14	0.16 (0.05 - 0.25)	0.10 (0.02 - 0.19)
Multiple imputation	IPTW+IPCW	14	0.16 (0.05 - 0.28)	0.12 (0.02 - 0.23)
